# An age-structured spatially varying coefficient model for high-resolution mapping of vaccination coverage

**DOI:** 10.1371/journal.pcbi.1013989

**Published:** 2026-02-17

**Authors:** C. Edson Utazi, Somnath Chaudhuri, Oghenebrume Wariri, Iyanuloluwa D. Olowe, Mohamed Megheib, Andrew J. Tatem

**Affiliations:** 1 WorldPop, School of Geography and Environmental Science, University of Southampton, Southampton, United Kingdom; 2 Southampton Statistical Sciences Research Institute, University of Southampton, United Kingdom; 3 Nnamdi Azikiwe University, Awka, Nigeria; 4 CIBER of Epidemiology and Public Health (CIBERESP), Madrid, Spain; 5 Department of Infectious Disease Epidemiology, London School of Hygiene and Tropical Medicine, London, United Kingdom; 6 Vaccines and Immunity Theme, Medical Research Council Unit, The Gambia at London School of Hygiene and Tropical Medicine, Fajara, The Gambia; 7 Department of Mathematical Statistics, Faculty of Graduate Studies for Statistical Research, Cairo University, Cairo, Egypt; Queen Mary University of London, UNITED KINGDOM OF GREAT BRITAIN AND NORTHERN IRELAND

## Abstract

High-resolution maps of vaccination coverage are valuable for uncovering heterogeneities in coverage to inform vaccine delivery strategies. Coverage maps stratified by age can reveal additional heterogeneities in the timeliness of vaccination and critical immunity gaps among birth cohorts. Here, we propose a spatially varying coefficient model relying on a Bayesian approach for age-structured mapping of vaccination coverage using geolocated individual level household survey and geospatial covariate data. Our flexible modelling framework includes parameterizations capturing spatial (non-)stationarity in differences in coverage between age groups, as well as a modification to allow coverage mapping for single age points through the inclusion of a smoother over age. The proposed models are fitted using the INLA-SPDE approach implemented in the inlabru package in R. We choose between competing model parameterizations by examining their out-of-sample predictive performance via cross-validation and using Bayesian model choice criteria. The methodology is applied to age-structured mapping of measles vaccination coverage in Cote d’Ivoire using the 2021 Demographic and Health Survey. Our results reveal a significant delay in measles vaccination in the first year of life and substantial spatial differences in coverage by age, highlighting the need for targeted interventions to achieve equity and attain vaccine-derived immunity goals.

## Author summary

This study presents a new method to create spatially detailed, age-specific maps of vaccination coverage, with a focus on measles vaccination in Côte d’Ivoire. Unlike traditional national or provincial estimates, these maps show where children are being vaccinated on a fine geographical scale, including differences between age groups. This matters because children who miss their vaccines early in life can leave pockets of vulnerability that fuel disease outbreaks. We used data from a 2021 national health survey implemented in the country, combined with maps of local conditions such as how far families travel to access health care, living standards, and the environment. We then applied a modern statistical technique – an age-structured spatially varying coefficient model – that flexibly adjusts how age-related vaccine coverage varies across space, and which can estimate coverage for age groups and single age points. This enabled us to create maps that uncovered not just where overall vaccination is low, but where in specific age groups (like children under 15 months) it’s falling behind. The results revealed important delays in measles vaccination during early childhood and highlighted geographic areas with particularly low coverage. By showing gaps in both age and location, this approach enables health workers to target immunization efforts more precisely, helping ensure that more children have timely protection against measles and potentially other diseases.

## 1. Introduction

Vaccination coverage is an important metric for monitoring the performance of immunization programmes [1,2]. Accurate estimates of vaccination coverage are essential at global, national, and subnational levels to assess the relative effectiveness of vaccine delivery strategies such as routine immunization and campaigns [3,4], to guide and evaluate targeted interventions [5], and to track progress toward national and global immunization goals [1,6,7], among other applications. For certain vaccines such as the measles-containing vaccine, estimates of coverage by age are particularly valuable since achieving coverage targets across all age groups and geographic areas is integral to disease elimination strategies [8]. Such estimates enable the monitoring of the timeliness of vaccination [9,10], identification of key immunity gaps across birth cohorts and the design of catch-up vaccination activities [11–13], thereby equipping national immunization programmes with the evidence needed to develop and implement effective approaches to achieve and sustain disease elimination.

Despite their importance, reliable estimates of vaccination coverage remain challenging to obtain, particularly at fine geographic scales. Data for estimating coverage are usually obtained from administrative sources and household surveys. The quality of data available from these sources can vary greatly due to inherent limitations such as incomplete reporting, recall bias and inaccuracies in population denominators, with survey estimates of coverage often taken to be the “gold standard” [2]. However, survey estimates are mostly representative for large administrative areas such as the national and first administrative levels due to the operational costs and logistical challenges of conducting more intensive sampling to produce estimates for more granular levels. These large area estimates mask epidemiologically and programmatically important heterogeneities in coverage. In line with increasing emphasis on more granular coverage estimates, as encapsulated in global policy frameworks such as the Immunization Agenda 2030 [1], there have been growing efforts to estimate coverage at more operationally relevant geographic scales to better inform vaccination programming [14–18]. Furthermore, survey-based estimates are typically reported for standard age groups, as determined by relevant vaccination schedules [7]; and when surveys are conducted infrequently, these estimates often fail to capture critical immunity gaps in other eligible age groups.

Few published studies such as Utazi et al [17] and Takahashi et al [15] have developed approaches for age-structured, high-resolution mapping of vaccination coverage using geolocated survey data. Utazi et al [17] employed a *birth cohort approach*, fitting independent geostatistical models to predict measles vaccination coverage in children aged 9–11 months, 12–23 months and 24–35 months. However, their methodology provides no mechanism to exploit shared patterns - such as the underlying spatial structures - across the age groups, which could potentially improve coverage estimation. Moreover, because separate models were fitted for each age group, their approach may perform poorly in contexts where overall or cluster-level sample sizes for a given age group are small (see, for example, the data section), since this can result in greater uncertainties in the modelled estimates for the age group [19]. Takahashi et al [15] adopted a *single age approach*, using a generalized additive model to map measles vaccination coverage by age in the African Great Lakes region. Their method incorporates a smooth function of age, enabling estimation of coverage at single-month age points but not for broader age groups. Additionally, the inclusion of a smooth function of survey cluster-level geocoordinates in their model primarily captures broad spatial trends and may be inadequate for modelling structured spatial dependence [20].

Spatially varying coefficient (SVC) models [21,22] are particularly useful in spatial regression contexts when relationships between an outcome and certain covariates exhibit spatial non-stationarity. These models allow the regression coefficients associated with these covariates to vary across space, yielding spatial surfaces/maps for these, and are also suitable for capturing differences in space between the levels of a given categorical covariate in relation to an outcome (see methods section). Gelfand et al [21] introduced a Bayesian framework for SVC models and these have been used in ecological settings in both discrete [23] and continuous [22] spatial domains to uncover nuanced spatial variation in ecological processes such as bird species abundance and forest biomass. In particular, Finley [22] compared different SVC model specifications, including geographically weighted regression [24], using both simulated and real-world datasets. The study accounted for directional (anisotropic) and nonstationary dependence structures, demonstrating the flexibility of these models to capture complex spatial patterns in the data.

In this study, we develop a novel and flexible methodology for age-structured mapping of vaccination coverage. The proposed approach is based on a spatially varying coefficient model that captures spatial non-stationarity in the differences in coverage between age groups, with alternative model specifications characterizing different forms of age-related variation, and which also enable the estimation of coverage using the single age approach. Unlike previous work [17,19,25], the proposed models are fitted to individual level data, enabling more robust estimation of covariate-coverage relationships and potentially improving estimation in areas with few cluster-level observations. Model selection is based on Bayesian model choice criteria and using cross-validation to evaluate out-of-sample predictive performance. The methodology is applied to age-disaggregated mapping of the coverage of the first dose of the measles-containing vaccine (MCV1) in Cote d’Ivoire, using data from the 2021 Demographic and Health Survey [26]. Cote d’Ivoire was chosen as a case study due to being a priority country for an implementing/donor organization and has therefore been the focus of recent work [27].

## 2. Methods

**Ethics statement**

Ethical approval for the study was provided by the University of Southampton (application ID: 95488.A1).

### 2.1. Vaccination coverage data

Geolocated individual-level data on the coverage of MCV1 were obtained from the 2021 Cote d’Ivoire DHS (CDHS) [26]. The survey was designed to be representative at the national and first administrative (14 areas known as districts – S1 Fig) levels, and for urban and rural areas. The sampling design involved a random, stratified two-stage selection, with urban and rural areas of each of the 14 districts constituting separate sampling strata, yielding a total of 28 strata. The first stage involved selecting the primary sampling units known as clusters (or enumeration areas) from a sampling frame, while the second stage involved selecting households within each sampled cluster, with 28 households sampled per cluster. In all, a total of 539 clusters were included in the survey [26].

**Fig 1:**
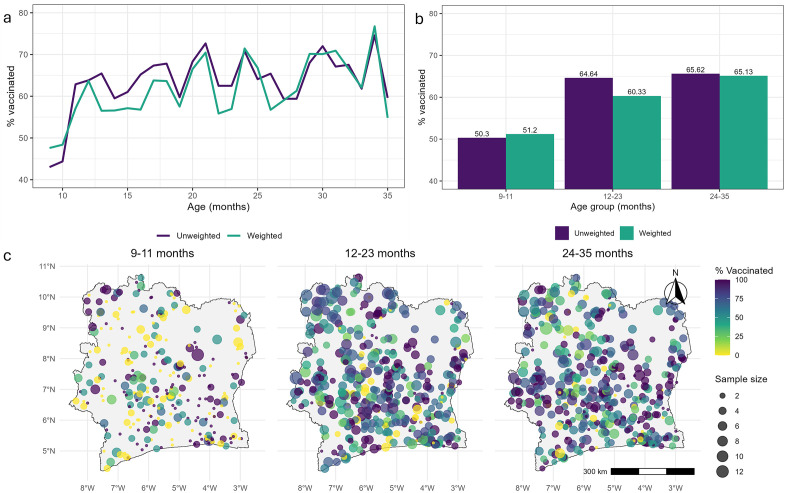
Empirical estimates of measles vaccination coverage for children aged 9 to 35 months at the national (a, b) and survey cluster (c) levels for (a) single age points and (b, c) age groups, according to the 2021 Cote d’Ivoire Demographic and Health Survey. Panels (a) and (b) show both survey weighted and unweighted estimates, whereas panel (c) shows unweighted estimates only. Boundary data used in panel (c) were obtained from geoBoundaries (www.geoboundaries.org) and are available under a CC BY 4.0 license.

Routine MCV1 is recommended for administration at 9 months of age in Cote d’Ivoire [28]. Hence, for each eligible child aged 9–35 months, we extracted information on their age, vaccination status (based on health records or caregiver recall) and the (latitude/longitude) geocoordinates of the cluster they were sampled from. Each individual level record was classified into one of three age groups - 9–11 months, 12–23 months or 24–35 months - representing different birth cohorts, and aggregated to the survey cluster level for validation purposes. The data extracted contained a total of 4,236 children, of whom 507, 1,920 and 1,809 belonged to the respective age groups. A summary of the extracted data is displayed in Figs 1 and S2. This includes national survey weighted and unweighted estimates of MCV1 coverage for single age points and age groups, unweighted coverage estimates at the cluster level for age groups and corresponding distribution of cluster level sample sizes for each age group.

These plots generally reveal a sharp increase in coverage at lower ages, which then appears to stabilize and exhibit random fluctuations in the later ages. The relatively lower coverage among children aged 9–11 months is evident both at the national and cluster levels. Cluster level sample sizes are much smaller for age 9–11 months, limiting the feasibility of a separate analysis for this age group. For the other age groups, cluster level sample sizes are also small (median = 3, interquartile range = 3). The distribution of these cluster-level sample sizes is particularly important for accurately estimating the underlying spatial structure in the data and for obtaining more robust point or grid level predictions of vaccination coverage across age groups.

### 2.2. Geospatial covariates, gridded population and boundary data

Following previous studies [3,17,19,25], we assembled a wide range of geospatial covariate datasets for the analysis. These include geographic, socioeconomic, remoteness, climate, environmental, conflict and health-related variables, which have either direct or proximate associations with vaccination coverage. While most of the covariates were externally sourced (see WorldPop [29] and S1 Table), others such as ownership of a health card/document were obtained from the 2021 CDHS [26]. These CDHS-derived covariates were first processed at the cluster level using the definitions provided in S1 Table. Kriging interpolation was then used to create their interpolated surfaces at 1x1 km resolution as outlined in previous work [19,30]. Covariate selection was undertaken in a non-spatial framework in R version 4.5.0 [31], using a similar approach as outlined in Utazi et al [17]. This involved examining the relationships between the covariates and vaccination coverage, and applying log transformations to the covariates where appropriate; fitting single-covariate models and ranking the covariates based on their predictive ability (using predictive R^2^ values); assessing multicollinearity and choosing between highly correlated covariates (with correlation > 0.8 or variance inflation factor > 4.0) based on their ranks; and finally, using stepwise regression (backward elimination based on the Akaike Information Criterion) to identify the best model or combination of covariates for modelling the outcome in a non-spatial framework with binomial regression models. A total of 13 covariates were selected for the analysis from an initial set of 38 covariates, including urban and rural areas, elevation, vegetation index (veg_index, average number of wet days (wetdays), urban accessibility (urban_access), elevation, maximum temperature (max_temp), distance to conflict areas (dist_conf), walking travel time to the nearest health facility (walking_tt), malaria prevalence (mal_prev), maternal education (mat_educ), health card/document ownership (health_card), access to media (media) and household wealth (wealth). Detailed definitions and sources of these covariates are provided in S1 Table. These selected covariates are displayed in S3 and S4 Figs.

Gridded population data for children aged under 5 years in 2021 were obtained from WorldPop [32]. These were used as weights when aggregating the grid level estimates to the administrative level [19]. All boundary data sets used in the analysis were obtained from geoBoundaries [33].

### 2.3. Model development

We begin by introducing the spatially varying coefficient model and its parameterizations for estimating vaccination coverage using the birth cohort approach, and then alternative specifications that enable estimation at single age points and for all eligible individuals.

Let ${\text{z}}_{i}(s_{j})$ denote the age (in months) of the ith eligible individual/child sampled from location $s_{j}$, and $a_{\text{0}},a_{\text{1}},\;a_{\text{2}},\ldots ,a_{K}$, the different K+1 age groups (or birth cohorts), one of which includes ${\text{z}}_{i}(s_{j})$. For example, in our application, K=2, and $a_{\text{0}}=\left\{\text{9},\;\text{1}\text{0},\;\text{1}\text{1}\text{ months}\right\};a_{\text{1}}=\{\text{1}\text{2},\ldots ,\;\text{2}\text{3}\text{ months}\}$ and $a_{\text{2}}=\{\text{2}\text{4},\ldots ,\;\text{3}\text{5}\text{ months}\}$. Also, let $Y_{i}\left(s_{j}\right)$ denote the corresponding binary outcome representing the individual’s vaccination status $\left(\text{i}.\text{e}.,\;Y_{i}\left(s_{j}\right)=\text{1}\text{ if vaccinated and }\text{0}\text{ otherwise}\right)$, and $p_{i}\left(s_{j}\right)$ the (unobserved) true probability of being vaccinated. The spatially varying coefficient model can be expressed as


$$Y_{i}\left(s_{j}\right)|p_{i}\left(s_{j}\right)\sim \text{Binomial}\left(\text{1},p_{i}\left(s_{j}\right)\right),\;i=\text{1},\ldots ,n_{j};j=\text{1},\ldots ,J,$$

$$\text{logit}\left(p_{i}\left(s_{j}\right)\right)=\beta_{\text{0}}+\sum {\mathrm{\backslash nolimits}}_{k=\text{1}}^{K}\left(\beta_{k}+\beta_{k}(s_{j})\right)I({\text{z}}_{i}(s_{j})\in a_{k})+𝐱{\left(s_{j}\right)}^{\prime }\alpha +\omega \left(s_{j}\right)+\in \left(s_{j}\right),$$ (1)

where $\beta_{\text{0}}$ is the overall intercept, $\beta_{k}$ and $\beta_{k}(s_{j})$ are fixed and spatially varying regression coefficients for age cohort $a_{k}(k>\text{0})$, I(.) is an indicator function that returns a value of 1 if the specified condition is true and 0 otherwise, and $𝐱(s_{j})$ is a vector of geospatial covariates for location $s_{j}$, with α being the corresponding fixed regression coefficients. Thus, $\beta_{k}(s_{j})$ can be viewed as a random spatial adjustment to the average change in the log-odds of vaccination for the kth age cohort, $\beta_{k}$, at location $s_{j}$. Note that the reference age group, $a_{\text{0}}$, is omitted from equation (1) as this is absorbed into the intercept terms. In practice, the choice of reference age group - the group against which others are compared - may vary depending on the objective of the analysis. Also, the covariate vector, $𝐱\left(s_{j}\right),\;$includes an urban-rural variable which helps to account for the urban-rural stratification used in the survey design. Only cluster-level geospatial covariates are included in the model since the goal of inference is to obtain age-specific coverage estimates at the grid level, typically at 1x1 km resolution.

Furthermore, the terms $\omega \left(s_{j}\right)$ and $\in (s_{j})$ are modelled as Gaussian random effects, capturing residual spatial and non-spatial (or between-cluster) variation, respectively. Specifically, $\omega \left(s_{j}\right)$ represents a random spatial adjustment to the overall intercept, $\beta_{\text{0}}$, such that $\beta_{\text{0}}+\omega \left(s_{j}\right)$ defines a spatially varying intercept process (for the reference age group). We model $\in (s_{j})$ as an independent and identically distributed (iid) process with mean 0 and variance $\sigma_{\in }^{\text{2}}$. The spatial random effect $\omega \left(s_{j}\right)$ is specified as a zero-mean Gaussian process with covariance function $\Sigma_{\omega }$, that is, $\omega ={\left(\omega \left(s_{\text{1}}\right),\ldots ,\omega (s_{J}\right)}^{\prime }\sim N(0,\;\Sigma_{\omega })$, where $\Sigma_{\omega }$ is assumed to follow the Matérn covariance function [34] given by


$$\Sigma_{\omega }\left(s_{j},{\;s}_{j^{\prime }}\right)=\frac{\sigma_{\omega }^{\text{2}}}{{\text{2}}^{\nu -\text{1}}\Gamma (\nu )}{\left(\kappa ∥s_{j}-s_{j^{\prime }}∥\right)}^{\nu }\;K_{\nu }\;\left(\kappa ∥s_{j}-s_{j^{\prime }}∥\right).$$

Here, the notation ∥.∥ denotes the Euclidean distance, $\sigma_{\omega }^{\text{2}}>\text{0}$ is the marginal variance of $\omega \left(s_{j}\right)$, κ>0 is a scaling parameter related to the range $r(r=\frac{\sqrt{\text{8}\nu }}{\kappa })$ – the distance at which spatial correlation is close to 0.1 - and $K_{\nu }$ is the modified Bessel function of the second kind and order ν>0. Following Lindgren et al [35], it is common to set the smoothness parameter ν equal to one to ensure identifiability.

For each of the spatially varying regression coefficients, $\beta_{k}\left(s_{j}\right),\;k=\text{1},\ldots K$, we also assume zero-mean Gaussian processes with Matérn covariance functions denoted by $\Sigma_{\beta_{k}}$. We denote the corresponding range and marginal variance parameters using $r_{k}$ and $\sigma_{\beta_{k}}^{\text{2}}$, respectively.

The general model in equation (1) can be further parameterized to yield simpler, more parsimonious models, depending on the nature of the dependencies assumed in the data. For example, if the spatially varying coefficients and the geospatial covariates are deemed sufficient to capture all the spatial dependencies, or more specifically, if the geospatial covariates are assumed to fully account for the spatial variation in the outcome for the reference age group, then model (1) reduces to


$$\text{logit}\left(p_{i}\left(s_{j}\right)\right)=\beta_{\text{0}}+\sum {\mathrm{\backslash nolimits}}_{k=\text{1}}^{K}\left(\beta_{k}+\beta_{k}(s_{j})\right)I({\text{z}}_{i}(s_{j})\in a_{k})+𝐱{\left(s_{j}\right)}^{\prime }\alpha +\in \left(s_{j}\right),$$ (2)

which excludes the spatially correlated residual term, $\omega \left(s_{j}\right)$. Other variants of model (2) are also possible. For instance, if prior evidence suggests that the step changes captured by $\beta_{k}$ are sufficient to characterise the differences between certain age groups and the reference age group, then the corresponding $\beta_{k}(s_{j})$ terms may also be excluded, while retaining $\omega \left(s_{j}\right)$. Such specifications may be particularly advantageous in contexts involving a large number of age groups.

Furthermore, if the differences in the odds of vaccination between each age group and the reference age group are assumed not to vary spatially and can be adequately characterised using fixed step changes, then the spatially varying coefficients can be removed entirely from model (1), yielding


$$\text{logit}\left(p_{i}\left(s_{j}\right)\right)=\beta_{\text{0}}+\sum {\mathrm{\backslash nolimits}}_{k=\text{1}}^{K}\beta_{k}I({\text{z}}_{i}(s_{j})\in a_{k})+𝐱{\left(s_{j}\right)}^{\prime }\alpha +\omega \left(s_{j}\right)+\in \left(s_{j}\right).$$ (3)

In practice, models (2) and (3) provide alternative model specifications against which to compare model (1) to determine the most appropriate modelling approach for capturing spatial dependencies in the data.

Additionally, when the goal is to predict coverage for single ages (in months), model (3) can be modified to include a smooth function of age, $f({\text{z}}_{i}(s_{j}))$:


$$\text{logit}\left(p_{i}\left(s_{j}\right)\right)=\beta_{\text{0}}+f\left({\text{z}}_{i}\left(s_{j}\right)\right)+𝐱{\left(s_{j}\right)}^{\prime }\alpha +\omega \left(s_{j}\right)+\in \left(s_{j}\right),$$ (4)

where f(.) is modelled using a second-order random walk: $f\left(z_{r}|z_{r-\text{1}},z_{r-\text{2}}\right)\sim N(\text{2}z_{r-\text{1}}-z_{r-\text{2}},\sigma_{z}^{\text{2}})$, which corresponds to the Bayesian equivalent of a cubic smoothing spline [36]. A sum-to-zero constraint was imposed on f(.) for identifiability, since the model includes an intercept term [36]. We note that, as in the birth cohort model, it is possible to model the effect of age in equation (4) using a spatially varying coefficient, such that $f\left({\text{z}}_{i}\left(s_{j}\right)\right)=\beta \left(s_{j}\right)z_{i}(s_{j})$. However, such parameterization is not ideal for estimating the overall smoothed effect of coverage by age as intended in model (4). Hence, model (4) captures the non-linear effect of age on vaccination but assumes constant residual spatial dependence for all ages as in model (3). Similar to model (3), model (4) can also be used to obtain predictions for age groups if desirable (see discussion section).

Finally, to predict coverage for all eligible age groups combined, the age-specific terms in model (1) can simply be dropped, yielding


$$\text{logit}\left(p_{i}\left(s_{j}\right)\right)=\beta_{\text{0}}+𝐱{\left(s_{j}\right)}^{\prime }\alpha +\omega \left(s_{j}\right)+\in \left(s_{j}\right).$$ (5)

Model (5) aligns with approaches commonly used in previous work to map vaccination coverage [16,19,25], except that it is estimated using individual rather than cluster level data. Note that in equation (5), as in all the models considered here, individual level variation is already accounted for in the binomial likelihood, with the variance of the logistic distribution fixed at $\frac{\pi^{\text{2}}}{\text{3}}≅\text{3}.\text{2}\text{9}$ [37].

For conciseness, we subsequently refer to the candidate birth cohort model specifications in equations (1) to (3) as MODsvc1, MODsvc2 and MODnosvc, respectively. Similarly, we refer to the single-age and all-age models in equations (4) and (5) as MODsmooth and MODall, respectively.

### 2.4. Bayesian inference, model fitting and prediction

The proposed models were implemented in a fully Bayesian framework. We placed the following prior distributions on the parameters:


$$\beta ={\left(\beta_{\text{0}},\;\beta_{\text{1}},\ldots ,\beta_{K}\right)}^{\prime }\;\sim N\left(0,{\text{1}\text{0}}^{\text{3}}I\right);\;\alpha \sim N\left(0,{\text{1}\text{0}}^{\text{3}}I\right);\;p\left(r<\tilde{r}\right)=\text{0}.\text{0}\text{1};\;p\left(r_{k}<\tilde{r}\right)=\text{0}.\text{0}\text{1};$$

$$p\left(\sigma_{\in }>\text{3}\right)=\text{0}.\text{0}\text{1};\;p\left(\sigma_{\omega }>\text{3}\right)=\text{0}.\text{0}\text{1};\;p\left(\sigma_{\beta_{k}}>\text{3}\right)=\text{0}.\text{0}\text{1};\;\sigma_{z}^{-\text{2}}\sim \text{Gamma}(\text{1},\;\text{0}.\text{01}).$$ (6)

These comprise non-informative priors on the fixed regression parameters and a weakly informative prior on the precision of the second-order random walk in MODsmooth. The priors on the range parameters and the remaining variance parameters are penalized complexity priors, introduced by Simpson et al [38] and used in Fuglstad et al [39] in a spatial modelling context, which penalize deviations from a base (simpler) model, e.g., a constant field. For the range parameters, $\tilde{r}$ is chosen to be 5% of the extent of the study area in the longer direction (north-south direction for the Cote d’Ivoire application).

Let $\theta =(\beta_{\text{0}},\beta_{\text{1}},\ldots ,\beta_{K},\alpha ,r,r_{\text{1}},\ldots ,r_{K},\sigma_{\in }^{\text{2}},\;\sigma_{\omega }^{\text{2}},\sigma_{\beta_{k}}^{\text{2}}{)}^{\prime }$ denote all the fixed parameters of MODsvc1 in equation (1), $\beta_{k}^{s}={\left(\beta_{k}\left(s_{\text{1}}\right),\ldots ,\beta_{k}(s_{J}\right)}^{\prime }$, $\in ={\left(\in \left(s_{\text{1}}\right),\ldots ,\in (s_{J}\right)}^{\prime }$ and y denote all the observed data. Its joint posterior distribution can be expressed as


$$\pi \left(\theta ,\omega ,\beta_{\text{1}}^{s},\ldots ,\beta_{K}^{s},\in |y\right)\propto \pi \left(\theta \right)\times N\left(\omega |0,\;\Sigma_{\omega }\right)\times \;N\left(\in |0,\;\sigma_{\in }^{\text{2}}I\right)\times N\left(\beta_{\text{0}}|\text{0},\;{\text{1}\text{0}}^{\text{3}}\right)$$

$$\times \prod {\mathrm{\backslash nolimits}}_{k=\text{1}}^{K}\left\{N\left(\beta_{k}^{s}|0,\;\Sigma_{\beta_{k}}\right)\times N\left(\beta_{k}|\text{0},\;{\text{1}\text{0}}^{\text{3}}\right)\right\}\;\;$$

$$\times \prod {\mathrm{\backslash nolimits}}_{j=\text{1}}^{J}\prod {\mathrm{\backslash nolimits}}_{i=\text{1}}^{n_{j}}\text{Binomial}(y_{i}\left(s_{j}\right);\;p_{i}\left(s_{j}\right),{\text{z}}_{i}(s_{j}),y),$$ (7)

where π(θ) is the joint prior distribution on θ. The posterior distributions of the other models can be straightforwardly derived from equation (7).

For parameter estimation, we employed the integrated nested Laplace approximation-stochastic partial differential equation (INLA-SPDE) approach, [35,40] implemented in the inlabru package in R version 4.5.0 [31]. The INLA approach performs approximate Bayesian inference for latent Gaussian models by using the Laplace method to approximate the marginal posterior distributions of all the unknown quantities in the model, including the parameters and latent variables. The SPDE approach facilitates the estimation of the spatial terms in the models (e.g., ω and $\beta_{k}^{s}$ in model (1)). The approach works by representing each of these terms as a Gaussian Markov random field to reduce the computational burden inherent in the estimation of their respective covariance matrices [35].

Of particular interest in the proposed models is the prediction of vaccination coverage for the age groups or single age points of interest at unsampled locations, e.g., 1×1 km grids, throughout the study area. In a Bayesian framework, the prediction of vaccination coverage for age group k∈{0,1,…,K} at a new location, $\tilde{s}$, proceeds by evaluating the posterior predictive distribution of $p_{k}\left(\tilde{s}\right)$ given by


$$\pi \left(p_{k}\left(\tilde{s}\right)|y\right)=\int \text{logi}{\text{t}}^{-\text{1}}\left(\pi \left(\eta_{k}\left(\tilde{s}\right)|\omega ,\in ,\beta_{k}^{s},\theta ,y\right)\right)$$

$$\times \pi \left(\omega ,\in ,\beta_{k}^{s},\theta |y\right)\;d\omega \;d\in \;d\beta_{k}^{s}\;d\theta ,$$ (8)

where $\eta_{k}\left(\tilde{s}\right)$ is the linear predictor for MODsvc1, such that


$$\eta_{k}\left(\tilde{s}\right)=\left\{ \begin{array}{c} @l\beta_{\text{0}}+𝐱{\left(\tilde{s}\right)}^{\prime }\alpha +\omega \left(\tilde{s}\right)+\in \left(\tilde{s}\right)\text{ if }k=\text{0}\\ \beta_{\text{0}}+\beta_{k}+\beta_{k}\left(\tilde{s}\right)+𝐱{\left(\tilde{s}\right)}^{\prime }\alpha +\omega \left(\tilde{s}\right)+\in \left(\tilde{s}\right)\text{ if }k\geq \text{1} \end{array}\right.\;.$$

In the inlabru package, equation (8) can be computed post model fitting by using either the generate() or predict() function. We obtained 1,000 posterior samples from equation (8) for each prediction grid location, which were then summarized to produce the grid level estimates and associated uncertainties. Administrative level estimates were also obtained as population-weighted averages over all the grid cells falling within each administrative unit [19], as noted previously, using these posterior samples. Individual level predictions (in- and out-of-sample prediction for validation purposes) and predictions using models (2), (3), (4) and (5) can also be obtained in a similar manner.

### 2.5. Model choice and validation

To compare the fits of the competing birth cohort models in equations (1) to (3) (and those of MODsmooth and MODall), we employed the Watanabe-Akaike Information Criterion (WAIC) [41,42] which uses the log of the predictive density of each data point to evaluate a model’s out-of-sample predictive performance. We note that a formal statistical comparison of the birth cohort models will be required in most applications, since the best model cannot always be determined *a priori* and to mitigate the risk of overfitting. The WAIC can be expressed as


$$WAIC=-\text{2}\sum {\mathrm{\backslash nolimits}}_{i=\text{1}}^{n}\text{log}\left\{\pi \left(y_{i}|y\right)\right\}+\text{2}p_{WAIC},$$

where $\pi \left(y_{i}|y\right)=\int_{-\infty }^{\infty }\pi \left(y_{i}|\theta \right)\pi \left(\theta |y\right)d\theta$ is the posterior predictive density of data point $y_{i}$ (out of n data points), which can be evaluated in practice as $\frac{\text{1}}{L}\sum_{l=\text{1}}^{L}\pi \left(y_{i}|\theta^{l}\right)$ with $\theta^{l}$ denoting the l-th posterior draw from a total of L posterior samples. The term $p_{WAIC}$ represents the effective number of parameters, computed as $p_{WAIC}=\sum_{i=\text{1}}^{n}Var_{\theta |y}[\text{log}(\pi (y_{i}|\theta ))]$. The WAIC balances model fit and complexity by penalizing overfitting through $p_{WAIC}$, which captures the sensitivity of the model’s fit to individual data points. A lower WAIC indicates better predictive accuracy.

To further evaluate the out-of-sample predictive performance of the proposed models both at the cluster and individual levels, we conducted a k-fold cross-validation exercise, creating the cross-validation folds as random and spatially stratified splits of J cluster locations and setting k=10. Under random cross-validation, clusters were randomly allocated to one of the k folds; in contrast, spatially stratified cross-validation assigned neighbouring cluster locations to the same fold. At the cluster level, we used the following metrics to evaluate predictive performance:


$$\text{Root mean square error},\;RMSE=\sqrt{\frac{\text{1}}{m}\sum {\mathrm{\backslash nolimits}}_{j=\text{1}}^{m}{\left(\hat{p}\left(s_{j}\right)-p\left(s_{j}\right)\right)}^{\text{2}}};$$

$$\text{Mean absolute error},\;MAE=\frac{\text{1}}{m}\sum {\mathrm{\backslash nolimits}}_{j=\text{1}}^{m}\left|\hat{p}\left(s_{j}\right)-p(s_{j})\right|;$$

$$\text{Average bias},\;AVG\_BIAS=\;\frac{\text{1}}{m}\sum {\mathrm{\backslash nolimits}}_{j=\text{1}}^{m}\left(\hat{p}\left(s_{j}\right)-p\left(s_{j}\right)\right);$$

Continuous ranked probability score,


$$CRPS=\frac{\text{1}}{m}\sum {\mathrm{\backslash nolimits}}_{j=\text{1}}^{m}\left\{\frac{\text{1}}{S}\sum {\mathrm{\backslash nolimits}}_{s=\text{1}}^{S}\left|{\hat{p}}^{\left(s\right)}\left(s_{j}\right)-p(s_{j})\right|-\frac{\text{1}}{\text{2}S^{\text{2}}}\sum {\mathrm{\backslash nolimits}}_{s=\text{1}}^{S}\sum {\mathrm{\backslash nolimits}}_{t=\text{1}}^{S}\left|{\hat{p}}^{\left(s\right)}\left(s_{j}\right)-{\hat{p}}^{\left(t\right)}(s_{j})\right|\right\};$$

where m is the number of cluster-level observations in the kth cross-validation set, $p\left(s_{j}\right)$ denotes the observed coverage and $\hat{p}\left(s_{j}\right)$ the corresponding predicted values (i. e., the posterior means). With the CRPS [43], the entire S posterior samples are used to measure the discrepancies between the observed values and the predicted values for each data point rather than using a particular summary as in the other metrics. The smaller the values of these metrics, the better the predictive performance.

At the individual level, we evaluated out-of-sample predictive performance by computing the Brier score, $BS=\frac{\text{1}}{M}\sum_{j=\text{1}}^{m}\sum_{i=\text{1}}^{n_{j}}{\left({\hat{p}}_{i}\left(s_{j}\right)-y_{i}\left(s_{j}\right)\right)}^{\text{2}}$, where $M=\sum_{j=\text{1}}^{m}n_{j}$ is the number of individual level observations in m validation set locations and $y_{i}(s_{j})$ denotes the binary outcomes [44]. A lower BS indicates better model performance. All the metrics were averaged over all k cross-validation folds in each case.

## 3. Results

### 3.1. Model choice

Table 1 presents the WAIC values of the candidate birth cohort models (MODsvc1, MODsvc2 and MODnosvc), along with those of MODsmooth and MODall for comparison. Among the three birth cohort models, MODsvc1 had the best predictive performance. All models that account for age clearly outperformed MODall, demonstrating better predictive accuracy at the individual level and substantial variation in coverage by age within the data. Notably, the better performance of MODsmooth relative to MODnosvc suggests that, in the absence of the spatially varying coefficients, the smooth age function in MODsmooth more effectively captured the gradients in age-specific coverage than the step changes in MODnosvc. Also, we observe that although MODsvc1 and MODsvc2 - both incorporating spatially varying coefficients - had the lowest WAIC, these also had the largest effective numbers of parameters relative to other models, as expected given their greater flexibility.

**Table 1:** WAIC values of the proposed models.

Model	${𝐩}_{WAIC}$	WAIC
MODsvc1	210	5191
MODsvc2	219	5197
MODnosvc	186	5215
MODsmooth	187	5204
MODall	184	5262

The patterns observed in the WAIC results are corroborated by the BS values reported in S2 Table. These results show that for individual level out-of-sample prediction under the random cross-validation scheme, MODsvc1 and MODsvc2 had the best predictive performance, although the differences between the models were modest. Under stratified cross-validation, the differences between the models were even smaller, with MODsvc1 still ranking among the best-performing models. Consistent with previous findings, for both cross-validation schemes, all models accounting for variation by age (except MODsvc2 under stratified cross-validation) outperformed MODall.

For out-of-sample prediction at the cluster level, Table 2 reports the validation metrics for MODsvc1, MODnosvc and MODsvc2, while S3 Table provides corresponding results for MODall for comparison. Due to sample size limitations, it was not feasible to evaluate cluster-level predictive performance for MODsmooth. Across the three models, out-of-sample predictive performance was broadly similar within each age group, though MODsvc1 performed slightly better overall, particularly for the 9–11 and 24–35 month age groups. For the 12–23 month age group, the differences between the models were marginal, with no model clearly outperforming the others. Importantly, all three models performed better for age groups 12–23 months and 24–35 months compared to 9–11 months (except when considering AVG_BIAS, which poorly differentiated between the models), likely reflecting the relatively larger cluster level sample sizes for both older age groups (Figs 1 and S2). Indeed, for the aggregated 9–35 month age group, the validation metrics reported in S3 Table are consistently lower than those in Table 2, indicating improved predictive performance associated with larger cluster level sample sizes. As expected, predictive performance across all the models was generally poorer under the stratified cross-validation scheme, reflecting the increased difficulty of prediction in this setting.

**Table 2:** Results of cluster level k-fold cross-validation.

Model	Age group	RMSE	MAE	AVG_BIAS	CRPS
**RANDOM**					
MODsvc1	9 -11	0.439	0.403	0.006	0.332
MODsvc2	9 -11	0.440	0.404	0.006	0.335
MODnosvc	9 -11	0.440	0.403	0.008	0.334
MODsvc1	12 - 23	0.315	0.263	0.008	0.206
MODsvc2	12 - 23	0.315	0.262	0.009	0.203
MODnosvc	12 - 23	0.314	0.262	0.012	0.208
MODsvc1	24 - 35	0.298	0.249	-0.015	0.197
MODsvc2	24 - 35	0.299	0.249	-0.015	0.196
MODnosvc	24 - 35	0.300	0.251	-0.016	0.198
**STRATIFIED**					
MODsvc1	9 - 11	0.444	0.408	0.004	0.337
MODsvc2	9 - 11	0.445	0.408	0.004	0.341
MODnosvc	9 - 11	0.445	0.409	0.004	0.338
MODsvc1	12 - 23	0.318	0.266	0.003	0.206
MODsvc2	12 - 23	0.320	0.267	0.002	0.207
MODnosvc	12 - 23	0.319	0.265	0.009	0.209
MODsvc1	24 - 35	0.304	0.252	-0.017	0.196
MODsvc2	24 - 35	0.302	0.253	-0.017	0.198
MODnosvc	24 - 35	0.304	0.255	-0.018	0.200

Overall, our evaluation of the out-of-sample predictive performance of the competing birth cohort models, at both the individual and cluster levels, indicates that MODsvc1 offers the best performance. Accordingly, in the following sections, we discuss the results from MODsvc1 when using the birth cohort approach to estimate vaccination coverage.

### 3.2. Parameter estimates

In Table 3, we present the estimates of the parameters of the best-fitting birth cohort model, MODsvc1. The covariates significantly associated with the odds of vaccination include: urban versus rural residence, distance to conflict areas, walking travel time to the nearest health facility, maternal education, health card/document ownership, access to media and age group. As expected, the odds of vaccination increased with greater distance to conflict areas, higher proportions of educated mothers, higher proportions of children who owned a health card and greater access to the media within households. Conversely, the odds of vaccination decreased as walking travel time to the nearest health facility increased.

**Table 3:** Table of parameter estimates. Estimates corresponding to significant regression coefficients are bolded.

Parameter	Mean	Odds ratio	Std. Dev.	2.5%	97.5%
${\hat{\beta }}_{\text{0}}$	-3.835	0.022	5.557	-14.727	7.057
Urban	**-0.611**	**0.543**	**0.143**	**-0.892**	**-0.33**
Veg_index	-3.384	0.034	2.143	-7.585	0.816
Wetdays	-0.102	0.903	0.102	-0.303	0.099
Dist_conf	**0.214**	**1.239**	**0.091**	**0.035**	**0.393**
Elevation	$-\text{1}\times {\text{1}\text{0}}^{-\text{5}}$	1.000	$\text{1}\times {\text{1}\text{0}}^{-\text{5}}$	$-\text{3}\times {\text{1}\text{0}}^{-\text{5}}$	$\text{1}\times {\text{1}\text{0}}^{-\text{5}}$
Urban_access	$-\text{2}\times {\text{1}\text{0}}^{-\text{4}}$	1.000	$\text{5}\times {\text{1}\text{0}}^{-\text{4}}$	-0.001	0.001
Walking_tt	**-0.002**	**0.998**	**0.001**	**-0.004**	**-0.001**
Mal_prev	-0.558	0.572	0.834	-2.192	1.077
Max_temp	0.117	1.124	0.144	-0.166	0.4
Mat_educ	**1.035**	**2.815**	**0.236**	**0.572**	**1.498**
Health_card	**1.824**	**6.197**	**0.302**	**1.231**	**2.417**
Media	**0.552**	**1.737**	**0.272**	**0.020**	**1.085**
Wealth	-0.135	0.874	0.247	-0.619	0.349
${\hat{\beta }}_{\text{1}(\text{1}\text{2}-\text{2}\text{3}\text{ months})}$	**0.716**	**2.046**	**0.124**	**0.473**	**0.959**
${\hat{\beta }}_{\text{2}(\text{2}\text{4}-\text{3}\text{5}\text{ months})}$	**0.827**	**2.286**	**0.305**	**0.228**	**1.425**
$\hat{r}$ (spatial range for ω)	0.844	–	0.456	0.293	2.028
${\hat{\sigma }}_{\omega }$ (marginal std. dev. for ω)	0.367	–	0.094	0.216	0.583
${\hat{r}}_{\beta_{\text{1}}}$(spatial range for $\beta_{\text{1}})$	0.702	–	0.344	0.283	1.595
${\hat{\sigma }}_{\beta_{\text{1}}}$(marginal std. dev. for $\beta_{\text{1}})$	0.399	–	0.112	0.214	0.649
${\hat{r}}_{\beta_{\text{2}}}$ (spatial range for $\beta_{\text{2}}$)	6.697	–	5.659	1.561	21.783
${\hat{\sigma }}_{\beta_{\text{2}}}$(marginal std. dev. for $\beta_{\text{2}}$)	0.400	–	0.17	0.171	0.828
${\hat{\sigma }}_{\in }^{-\text{2}}$ (precision for ∈)	4.056	–	1.228	2.151	6.94

Considering the estimated odds ratios, a unit increase in the proportion of children who owned a health card is associated with a sixfold increase in the odds of vaccination, for example. Notably, children residing in urban areas had 46% lower odds of being vaccinated compared to those in rural areas – a pattern that appears to be specific to the study country [26]. Furthermore, on average, children aged 12–23 months and 24–35 months had approximately twice the odds of being vaccinated compared to those aged 9–11 months, suggesting significant delays in MCV1 vaccination during the first year of life.

The spatial variation in the differences in odds of vaccination between the age groups is illustrated by the maps of $\beta_{\text{1}}(s)$ and $\beta_{\text{2}}(s)$ in S5 Fig. The estimated surface for $\beta_{\text{2}}(s)$ appears much smoother than that for $\beta_{\text{1}}(s)$, suggesting greater spatial variability in the differences (in the log-odds of vaccination) between age groups 9–11 months and 12–23 months than between 9–11 months and 24–35 months. This provides a strong justification for the use of spatially varying coefficients in our modelling approach, but it also indicates that to preserve parsimony in the model, the difference in the likelihood of vaccination between ages 9–11 months and 24–35 months could be modelled as a step change, as discussed previously in the methods section. We discuss the spatial patterns in the differences in coverage between the age groups in detail in Section 3.3.

The estimated spatial ranges for the spatially varying coefficients - $\beta_{\text{1}}(s)$ and $\beta_{\text{2}}(s)$ - are 78 km and 743 km, respectively, while that of the spatial random effect, ω, is 94 km. Again, these estimates suggest much greater spatial heterogeneities in the differences in coverage between age groups 9–11 months and 12–23 months than between 9–11 months and 24–35 months. Nevertheless, the corresponding marginal variance estimates for these terms are very close, and the iid residual term, ∈, accounts for nearly twice as much variation in the model as ω.

Similar patterns are observed in the results of MODsmooth reported in S4 Table and S6 Fig, both in terms of significant covariate effects and the effect of age on vaccination. In particular, the delay in the receipt of MCV1 is evident in the estimated nonlinear effect of age on vaccination, which shows a markedly lower probability of vaccination among children aged < 15 months relative to older ages. We observe a steep increase in the probability of vaccination from age 9 months to about 15 months, which then stabilizes at a high level with minor fluctuations beyond 23 months, suggesting immunity gaps in the 24–35 month birth cohort. The credible intervals for the smooth functions indicate greater uncertainty beyond age 12 months due to greater fluctuations in coverage at older ages (see Fig 1a).

### 3.3. Predicted 1x1 km maps of vaccination coverage for age groups and single age points

**Fig 2:**
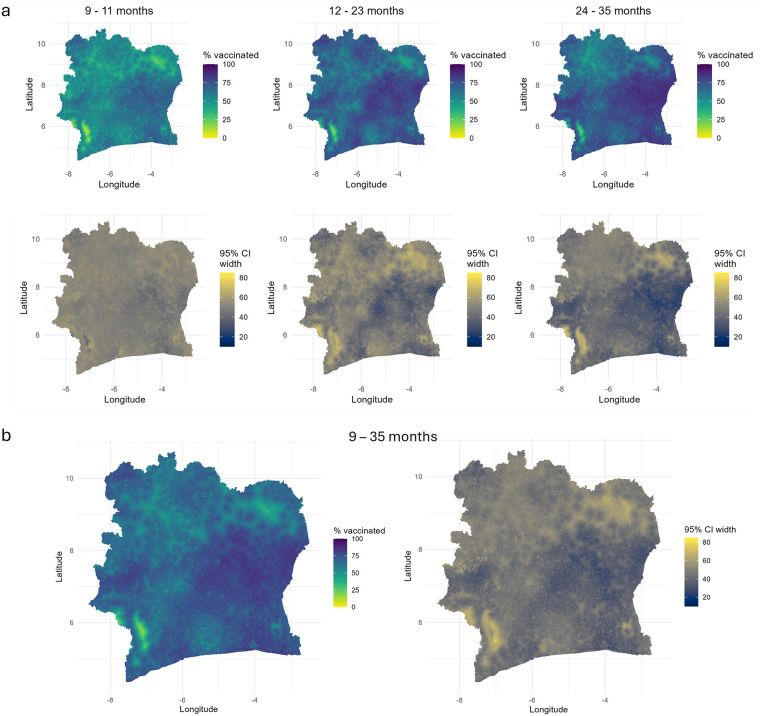
1x1 km maps of MCV1 coverage and associated uncertainties (95% credible interval widths) for age groups 9-11 months, 12-23 months, 24-35 months (panel a) and 9-35 months (panel b).

Geographically, considerable heterogeneities in MCV1 coverage are observed across all age groups (Fig 2), with a pronounced effect of walking travel time to the nearest health facility (see S4 Fig). The 1x1 km maps generally reveal lower coverage in the 9–11 month age group relative to other age groups, particularly concentrated in the western and northern districts of the country. The spatial distributions of coverage in these age groups show some interesting similarities, but there are also striking differences. Across all the age groups, coverage levels are consistently higher in and around Lacs, Vallée du Bandama and Yamoussoukro districts. Whilst overall coverage levels do not differ substantially between ages 12–23 months and 24–35 months, the spatial patterns in coverage appear to be more similar between ages 9–11 months and 24–35 months than 12–23 months, with the latter showing more pronounced spatial gradients, as noted previously. The modelled estimates at the national level are 54.1%, 67.9% and 70.9% for the respective age groups. Compared to corresponding direct survey estimates of 51.2%, 60.3% and 65.1%, the model slightly overestimated coverage, especially in the 12–23 month age group. For the combined 9–35 month age group, similar spatial patterns are observed, although aggregating over this wider age range obscures differences in coverage between birth cohorts and is more suitable when interest is in estimating for broader age bands.

The widths of the 95% credible intervals (CIs) associated with the coverage maps indicate greater uncertainties in areas with sparse survey data (e.g., the Zanzan region; see Fig 1) and in areas of lower coverage, especially for age groups 12–23 months and 24–35 months. Uncertainties for the 9–11 month group show less spatial variation but are slightly higher overall, likely reflecting smaller sample sizes. The median widths of the 95% CIs are 50%, 48%, and 43% for the 9–11, 12–23, and 24–35 month groups, respectively, indicating considerable uncertainties in the grid-level estimates.

**Fig 3:**
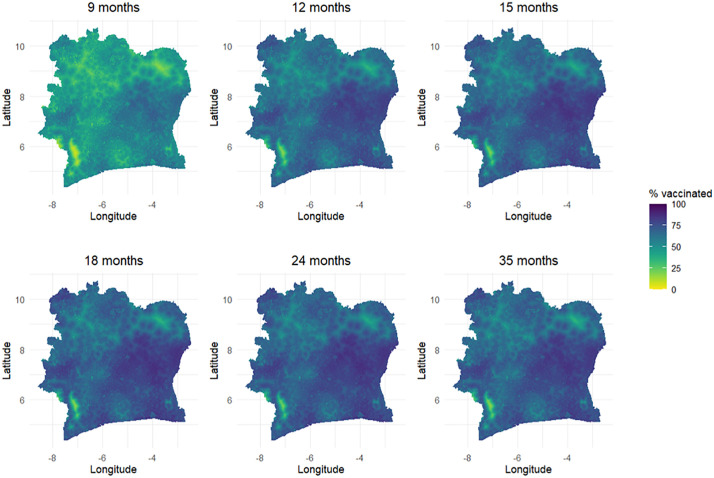
1x1 km maps of MCV1 coverage for single age points at 9, 12, 15, 18, 24 and 35 months. Corresponding uncertainty estimates, obtained as the widths of the 95% credible intervals, are shown in the supplementary file.

At single age points (Fig 3), lower coverage at 9 months is also evident, with very similar spatial patterns observed from ages 12–35 months. (Note that MODsmooth does not capture spatial gradients in the differences between age groups.) The modelled national coverage estimates of 47.6%, 65%, 68.3%, 70%, 70.3% and 68.7% at 9, 12, 15, 18, 24 and 35 months, respectively, further confirm that coverage patterns stabilize and become more similar from age 15 months and above. The corresponding uncertainties, reported in S7 Fig, show similar patterns and ranges as those in Fig 2.

### 3.4. Estimation of vaccination coverage at the administrative level

**Fig 4:**
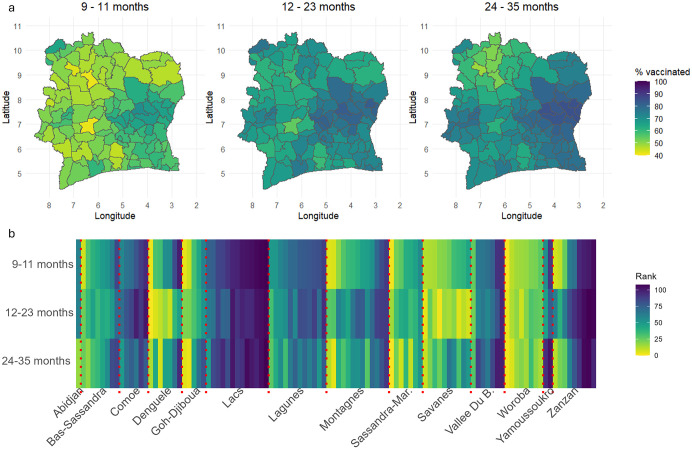
Departmental level estimates of MCV1 coverage. (a) Estimates of MCV1 coverage and (b) corresponding ranks at the departmental level for each age group. The dotted vertical lines in panel (b) differentiate between the departments in each of the 14 districts of Cote d’Ivoire. Boundary data used in panel (b) were obtained from geoBoundaries (www.geoboundaries.org) and are available under a CC BY 4.0 license.

At the departmental level, differences in both coverage and spatial prioritization for the 9–11, 12–23, and 24–35 month age groups are shown in Figs 4 and S8. Consistent with the national-level estimates reported earlier, coverage remains suboptimal at the departmental level, with no department reaching the target of 95% coverage in any age group. In particular, for the 9–11 month age group, coverage is estimated to be below 60% in about 70% of departments. For the 24–35 month age group, clusters of departments with lower coverage (<60%) are observed in the neighboring Savanes (M’Bengué, Tengrela, Boundiali, and Kouto) and Denguélé (Kaniasso, Madinani, and Séguélon) districts, despite overall higher coverage at older ages - potentially contributing to the dip in coverage after 23 months noted in S6 Fig.

Among 9–11 month olds, the highest coverage levels (>67%) are estimated in M’Bahiakro, Daoukro, and Prikro (all in the Lacs district), while the lowest (<44%) are in Dianra, Daloa, and Séguélon departments. For 12–23 months, coverage is highest (>81%) in M’Bahiakro, Tanda, and Transua (Lacs and Zanzan districts), and lowest (<47%) in Daloa, Dianra, and Guitry. In the 24–35 month group, coverage peaks (>85%) in Tanda, Koun-Fao, M’Bahiakro, and Transua (Zanzan and Lacs districts), and is lowest in Séguélon, Kouto, and Dianra. Although there are similarities in the locations of higher and lower coverage across age groups, differences also emerge, as reflected in the departmental rankings shown in Fig 4b. For example, even within Lacs district - where coverage is consistently higher across all age groups – there are differences in the ranks of departments when comparing the age groups. The rankings of the 9–11 and 24–35 month groups are generally more similar, corroborating earlier findings about the resemblance in their spatial coverage patterns. The median absolute differences in departmental ranks between age groups are: 9 (IQR = 12) for 9–11 vs. 12–23 months, 8 (IQR = 11) for 9–11 vs. 24–35 months, and 14 (IQR = 18) for 12–23 vs. 24–35 months. Furthermore, the rankings appear to vary most in the Zanzan, Denguélé, and Montagnes districts (based on the larger IQRs of ranks across all age groups within these districts).

The uncertainties associated with the departmental-level estimates (S9 Fig) are considerably smaller than those of the grid-level estimates, with median 95% CI widths of 23%, 22%, and 18% for the respective age groups.

We also estimated coverage at the district level for the 12–23 month age group to enable comparison with direct survey estimates at this level (S10 Fig). Overall, there is good agreement between the modelled and direct survey estimates; however, the relative overestimation of coverage observed earlier is most pronounced in the Abidjan, Comoé, and Lagunes districts.

## 4. Discussion

Our study proposed and evaluated a spatially varying coefficient model for age-structured mapping of vaccination coverage, along with alternative specifications offering the flexibility to map coverage for both age groups and single age points and intrinsically guarding against overfitting. The methodology is also applicable to mapping other health and development indicators where understanding age-related differences is of interest. The proposed models can be readily implemented using the INLA-SPDE approach, although predictions at a high resolution (e.g., 1 × 1 km) and over large study areas may require high-memory computing environments.

Among the three competing birth cohort models, our validation results based on the 2021 CDHS data provided sufficient support for the spatially varying coefficient model, MODsvc1. We also found that explicitly modelling age improved predictive performance at the individual level. As expected, however, out-of-sample prediction across multiple age categories is constrained by smaller sample sizes compared to predictions for all ages combined. The impact of cluster-level sample sizes on out-of-sample prediction has been explored in detail elsewhere [19]. For larger age groups, more parsimonious parameterizations of MODsvc1 - incorporating fixed step changes between certain age groups - can be informed by the data, guided by model selection criteria, or refined through inspection of the predicted surfaces from the fully parameterized model and subsequent adjustments in later analyses.

The estimated covariate effects in our analysis highlight the need for interventions aiming to improve coverage in urban areas, conflict areas, areas with limited access to health facilities/vaccination services, as well as those targeted towards improved maternal/caregiver education, improved vaccination card retention (also an indication of improved access to vaccination services) and improved vaccine information communication in Cote d’Ivoire. These findings are corroborated by previous studies [45,46] which analysed similar indicators of vaccination coverage in Cote d’Ivoire. Our study also demonstrated a profound effect of age on vaccination. In the birth cohort model MODsvc1, we estimated that children aged 12–23 months and 24–35 months had approximately twice the odds of being vaccinated compared to those aged 9–11 months. We also observed substantial spatial heterogeneity in the differences in coverage between the 9–11 and 12–23 month age groups. In the single-age model MODsmooth, we found markedly lower vaccination probabilities among children younger than 15 months, as well as slight dips in coverage within the 24–35 month cohort. Together, these findings provide strong evidence of significant delays in MCV1 vaccination during the first year of life. If these patterns persist, interventions targeting eligible children during the first year of life and up to 15 months (e.g., improved maternal/caregiver education and improved access to vaccination services) are likely to be most impactful.

Our predicted maps clearly showed that MCV1 coverage is lowest at younger ages (around the first year of life) in the northern and western districts, and further corroborated the relatively higher and more uniform coverage at older ages. These spatial patterns in coverage are also consistent with Ahoussou et al’s [47] study, which analysed measles case-based surveillance data in Cote d’Ivoire and found relatively higher odds of detection of positive measles-specific immunoglobin M (IgM) in health regions in the west of the country. At the grid, departmental, and district levels, estimated coverage remained well below the 95% target across the country. Specifically, in the 9–11 month age group, coverage was estimated to be below 60% in about 70% of departments. In the 24–35 month age group, despite generally higher coverage, areas of lower coverage (<60%) were concentrated in the neighboring Savanes (M’Bengué, Tengrela, Boundiali, and Kouto) and Denguélé (Kaniasso, Madinani, and Séguélon) districts. Across all age groups, we identified notable differences in departmental rankings, highlighting distinct priority areas for each age group, although some consistent patterns also emerged. For example, the departments of Dianra (Woroba district), Daloa (Sassandra–Marahoué), and Séguélon (Denguélé) were consistently among the three lowest-coverage areas in at least two age groups, whereas Guitry and Kouto were among the lowest-coverage areas only for the 12–23 and 24–35 month groups, respectively. Departmental rankings were most variable in the Zanzan, Denguélé, and Montagnes districts, indicating greater heterogeneity in age-specific coverage within these regions. The uncertainties in these modelled estimates were higher at the grid level (with median 95% CI widths ranging between 43% and 50%) than at the departmental level (with median 95% CI widths ranging between 18% and 23%), suggesting that departmental-level estimates are more actionable for programmatic purposes. Nevertheless, producing estimates at the grid level first provides the flexibility to aggregate to operationally relevant spatial scales where precision improves, while retaining the spatial granularity necessary for targeted interventions.

Our results have several implications for measles control and elimination efforts in Cote d’Ivoire. WHO and UNICEF estimates indicate that national MCV1 coverage remained suboptimal at 70% in 2023 [48]. In 2024 alone, Cote d’Ivoire reported 1,872 measles cases to WHO - a 37.5% increase from the previous year [49]. Addressing existing immunity gaps is therefore critical for progress towards measles control and elimination, and our study provides evidence to help guide intervention strategies. First, our modelled estimates for the 9–11 month age group can support routine immunization interventions aimed at improving coverage in the first year of life, or in children up to 15 months of age, by identifying and prioritizing the areas most at risk, as has been recommended in other settings [50,51]. Second, these estimates can inform the planning of catch-up vaccination activities, particularly for children currently aged 5–9 years, which encompasses two of the birth cohorts analyzed in our study (12–23 months and 24–35 months at the time of the survey). For spatial prioritization, the rankings of subnational areas, as demonstrated in Fig 4, could be enhanced by using estimates of the number of zero-dose children, which may better capture areas of highest disease risk. These zero-dose estimates can also feed into microplanning for vaccination activities, while field data collected during such activities can in turn be used to validate and refine the modelled estimates.

Our findings could be further strengthened through triangulation with measles case-based surveillance data or serosurveillance data that reflect the national immunity profile, helping to build a more robust evidence base for targeted interventions [25,47,52]. On the technical side, the spatially varying coefficient model developed here can be extended to age-dependent analyses of these aggregate programmatic data at the district or provincial level [23]. Such extensions will typically involve modelling the spatially varying regression coefficients and residual spatial dependence in the model using conditional autoregressive priors [53] and considering alternative probability distributions for the outcome. Additionally, it is possible to adapt MODsmooth to produce estimates over a specified age range. This will typically involve computing $\frac{\text{1}}{z_{\text{2}}-z_{\text{1}}}\int_{z_{\text{1}}}^{z_{\text{2}}}f(z)dz$ by defining a fine grid within the interval ${[z}_{\text{1}},\;z_{\text{2}}]$, computing the posterior mean of f(z) at each grid point and then averaging over these points. In R-INLA, the integration points over $\left[z_{\text{1}},\;z_{\text{2}}\right]$ can be included in f(.) during model fitting and then the marginal posterior distributions of f(.) can be obtained post model-fitting and averaged to compute the integral. However, as noted earlier, this approach does not account for spatial non-stationarity when estimating differences in coverage between the age groups. Moreover, estimating age effects at single age points using a spatially varying coefficient model is not well suited for capturing the overall smoothed effect of age (see S11 Fig). Indeed, in our 2021 CDHS application, such a specification underperformed (WAIC = 5232) relative to MODsmooth in equation (4). In future work, we plan to explore spatiotemporal extensions of the proposed models to allow age- and time-specific estimation of coverage. We anticipate that this analysis will yield additional programmatic insights into how the timeliness of vaccination and differences in coverage between the age groups have changed over space and time within and across countries.

Our analysis has some limitations. In the CDHS application, small cluster-level sample sizes - particularly in the 9–11 month age group - resulted in greater uncertainty in the predictions. We also observed that the fitted model slightly overestimated coverage in some southeastern areas of the country, potentially due to limited sample sizes, poor spatial coverage of clusters in these areas, or oversmoothing by the model. Furthermore, our analysis relied on information from both caregiver recall and vaccination records, which could introduce recall bias. The age range considered in our application was also constrained by data availability – DHS surveys only collect information on vaccination coverage for children aged 0–35 months at the time of the survey. As noted earlier, analyses covering broader age ranges are likely to yield greater insights for program planning and decision-making.

In summary, our study demonstrates the value of the proposed spatially varying coefficient model and alternative specifications for producing high-resolution maps of vaccination coverage by age and identifying priority areas for interventions. By highlighting substantial geographic and age-related heterogeneities in MCV1 coverage in our application, our findings underscore the need for targeted strategies to close immunity gaps, particularly in younger children and underserved areas. The proposed methodological framework is flexible, scalable, and applicable to other health indicators, offering a useful tool to support evidence-based decision-making for immunization programs and beyond. Future extensions to the spatiotemporal setting will further enhance its utility.

## Supporting information

S1 Fig A map of the 14 districts of Cote d’Ivoire comprising the first administrative level. Boundary data used in the plot were obtained from geoBoundaries (www.geoboundaries.org) and are available under a CC BY 4.0 license.
(DOCX)

S2 Fig Histograms of cluster-level sample sizes for different age groups considered in the study. (DOCX)

S3 Fig Some of the geospatial covariates selected for the analysis. (DOCX)

S4 Fig Some of the geospatial covariates selected for the analysis. (DOCX)

S5 Fig Maps of β_1 (s) and β_2 (s) capturing random spatial adjustments to differences in (the log-odds of) vaccination between the 9-11 month age group and each of 12-23 month and 24-35 month age groups, respectively, estimated using MODsvc1. (DOCX)

S6 Fig Plots of the estimated smooth function of age and associated uncertainties using MODsmooth on the (a) logit (i.e., log-odds) and (b) probability scales. (DOCX)

S7 Fig Uncertainty maps (95% credible interval (CI) widths) associated with 1x1 km estimates of MCV1 coverage for single age points. (DOCX)

S8 Fig Department level estimates of MCV1 coverage and associated uncertainties (95% credible intervals) for each of the 14 districts of Cote d’Ivoire. The plots have been ordered according to the estimated coverage in the 9-11 month age group.
(DOCX)

S9 Fig Uncertainty (95% credible interval width) maps for department level estimates of MCV1 coverage for all three age groups. Boundary data used in these plots were obtained from geoBoundaries (www.geoboundaries.org) and are available under a CC BY 4.0 license.
(DOCX)

S10 Fig Direct versus modelled estimates of MCV1 coverage for age group 12-23 months. (DOCX)

S11 Fig Overall smoothed effect of age when using a spatially varying coefficient to model the effect of age for single age points (in MODsmooth). (DOCX)

S1 Table Descriptions of the geospatial covariates used in the study. Covariates 1 – 9 are the externally sourced geospatial covariates while covariates 10 – 13 are the DHS-derived covariates.
(DOCX)

S2 Table Brier scores of the fitted models. (DOCX)

S3 Table K-fold cross-validation results for MODall (i.e., for age 9-35 months). (DOCX)

S4 Table Table of parameter estimates for MODsmooth. Estimates corresponding to significant regression coefficients are bolded.
(DOCX)
