# Supplementary material for: An age-structured spatially varying coefficient model for high-resolution mapping of vaccination coverage
Source: PLoS Comput Biol. 2026 Feb 17;22(2):e1013989. doi: 10.1371/journal.pcbi.1013989 (PMC12928601; doi:10.1371/journal.pcbi.1013989)
Supplement: S1 Fig — Boundary data used in the plot were obtained from geoBoundaries (www.geoboundaries.org) and are available under a CC BY 4.0 license. (DOCX) [file pcbi.1013989.s001.docx]

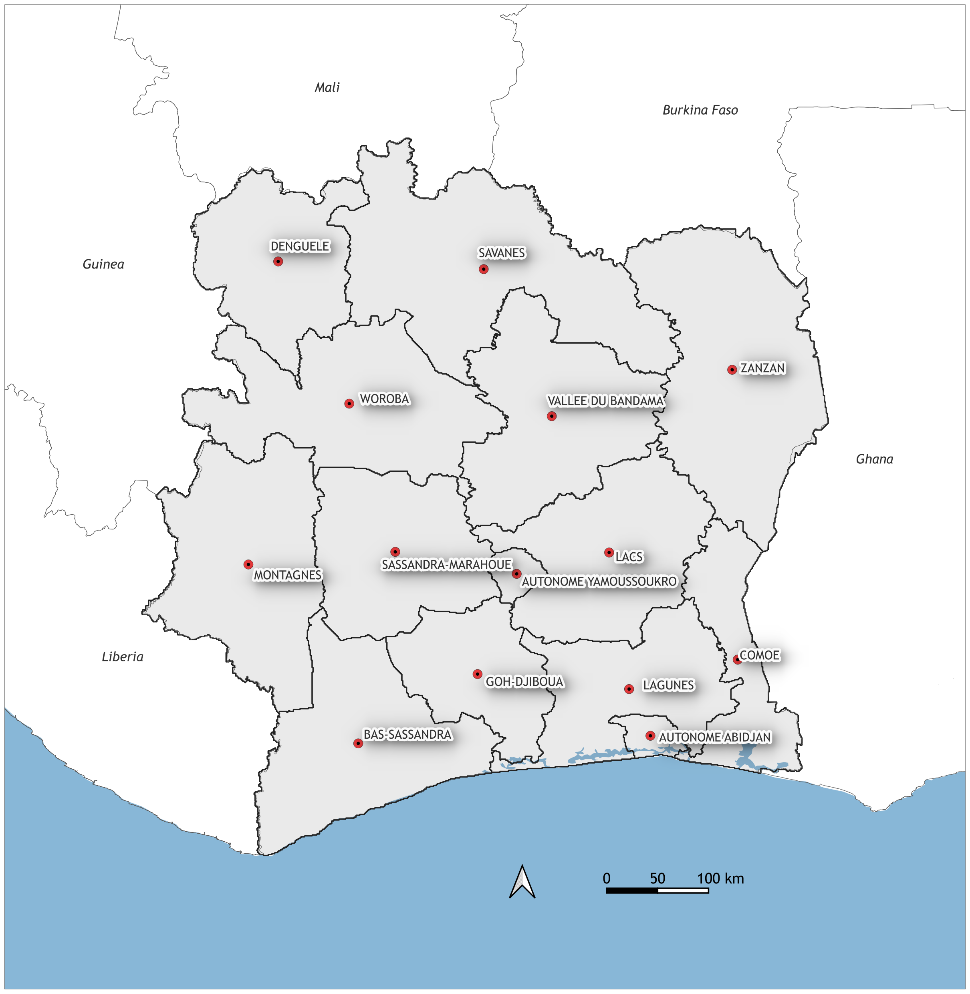


S1 Fig: A map of the 14 districts of Cote d’Ivoire comprising the first administrative level. Boundary data used in the plot were obtained from geoBoundaries ([www.geoboundaries.org](http://www.geoboundaries.org)) and are available under a CC BY 4.0 license.
