# Supplementary material for: An age-structured spatially varying coefficient model for high-resolution mapping of vaccination coverage
Source: PLoS Comput Biol. 2026 Feb 17;22(2):e1013989. doi: 10.1371/journal.pcbi.1013989 (PMC12928601; doi:10.1371/journal.pcbi.1013989)
Supplement: S2 Fig — (DOCX) [file pcbi.1013989.s002.docx]

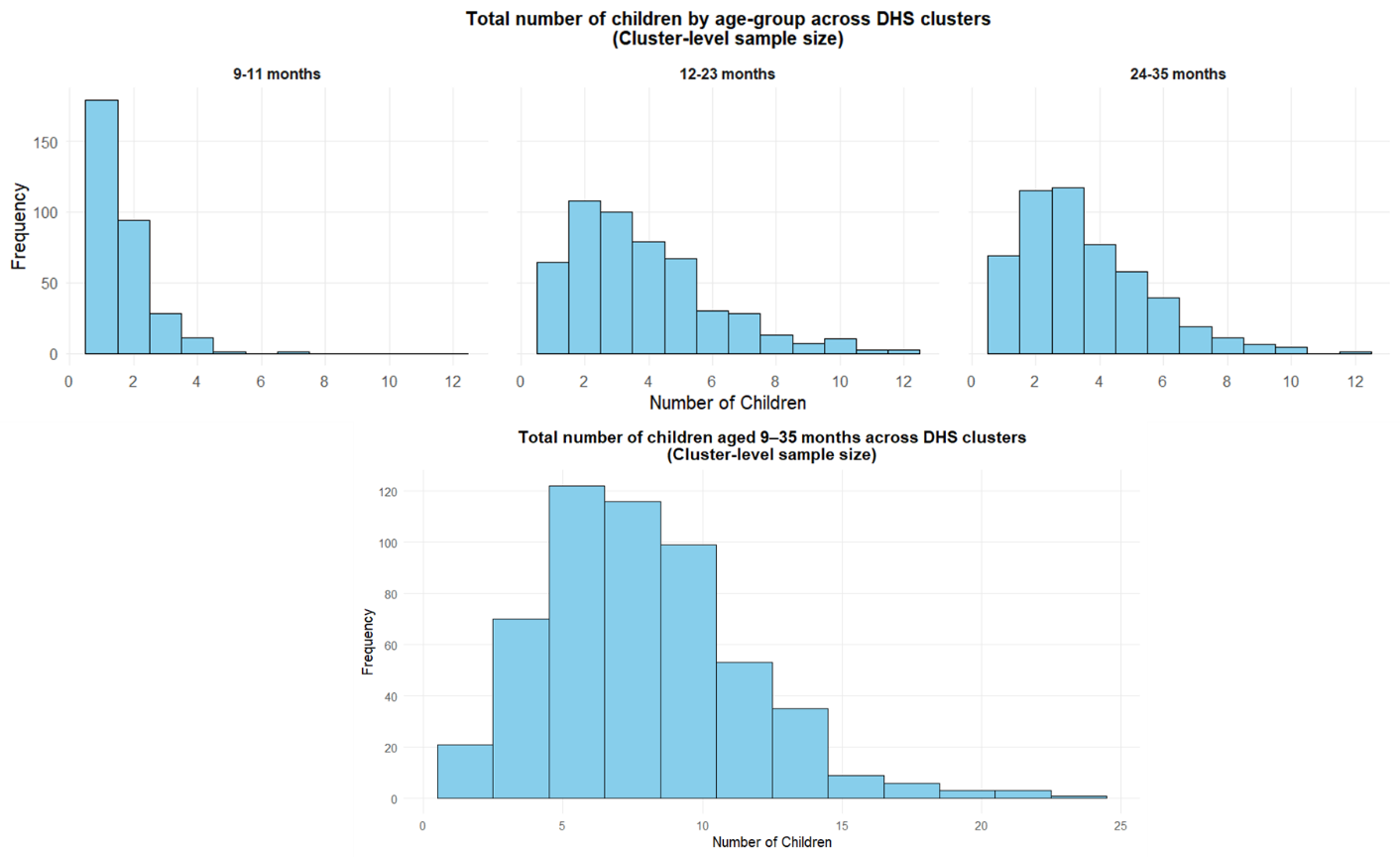


S2 Fig: Histograms of cluster-level sample sizes for different age groups considered in the study
