# Supplementary figures and images for: An age-structured spatially varying coefficient model for high-resolution mapping of vaccination coverage
Source: PLoS Comput Biol. 2026 Feb 17;22(2):e1013989. doi: 10.1371/journal.pcbi.1013989 (PMC12928601; doi:10.1371/journal.pcbi.1013989)

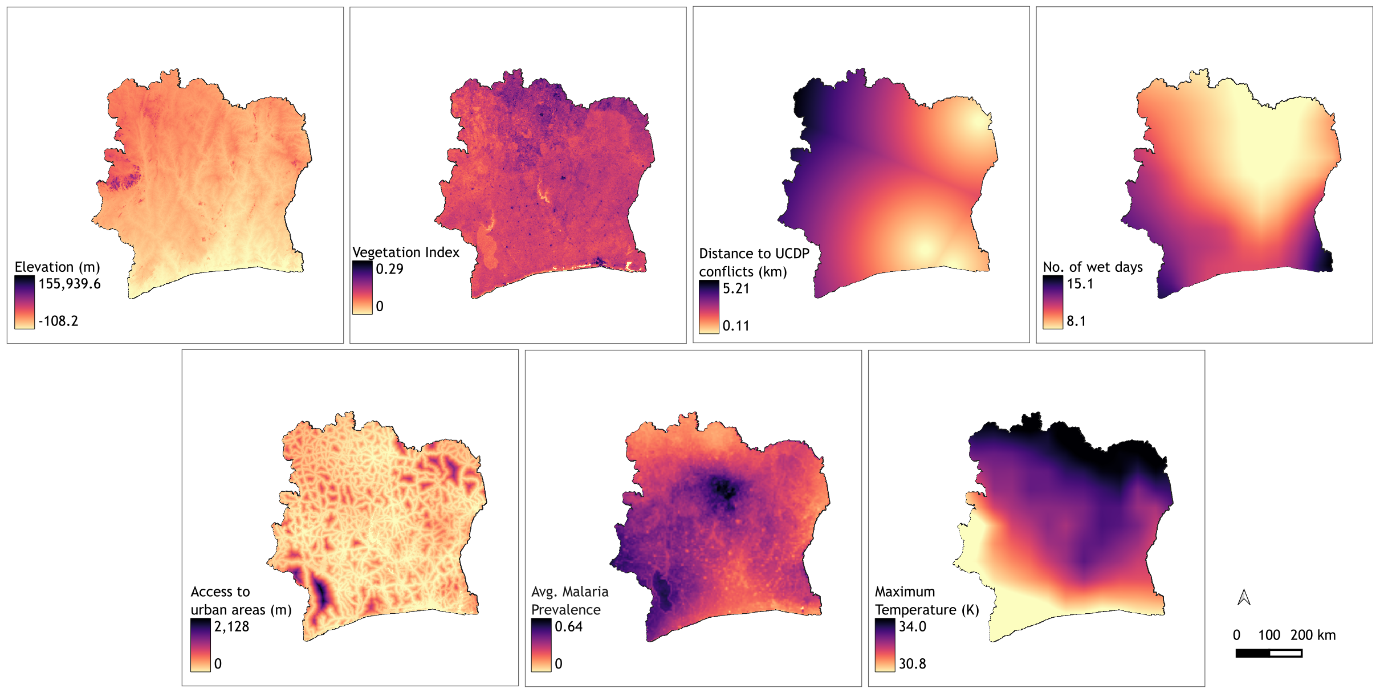


S3 Fig: Some of the geospatial covariates selected for the analysis.

Supplement: S3 Fig — (DOCX) [file pcbi.1013989.s003.docx]

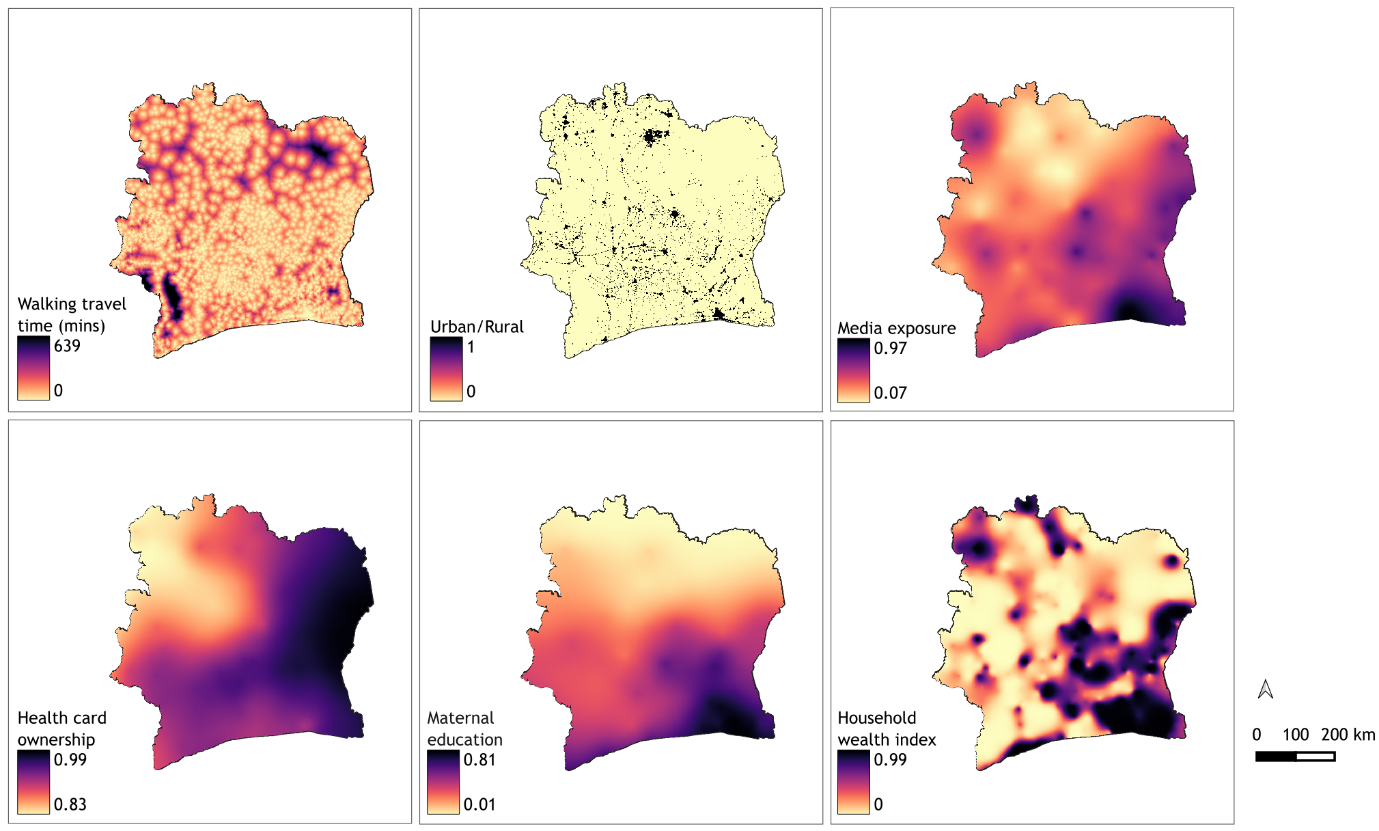


S4 Fig: Some of the geospatial covariates selected for the analysis.

Supplement: S4 Fig — (DOCX) [file pcbi.1013989.s004.docx]

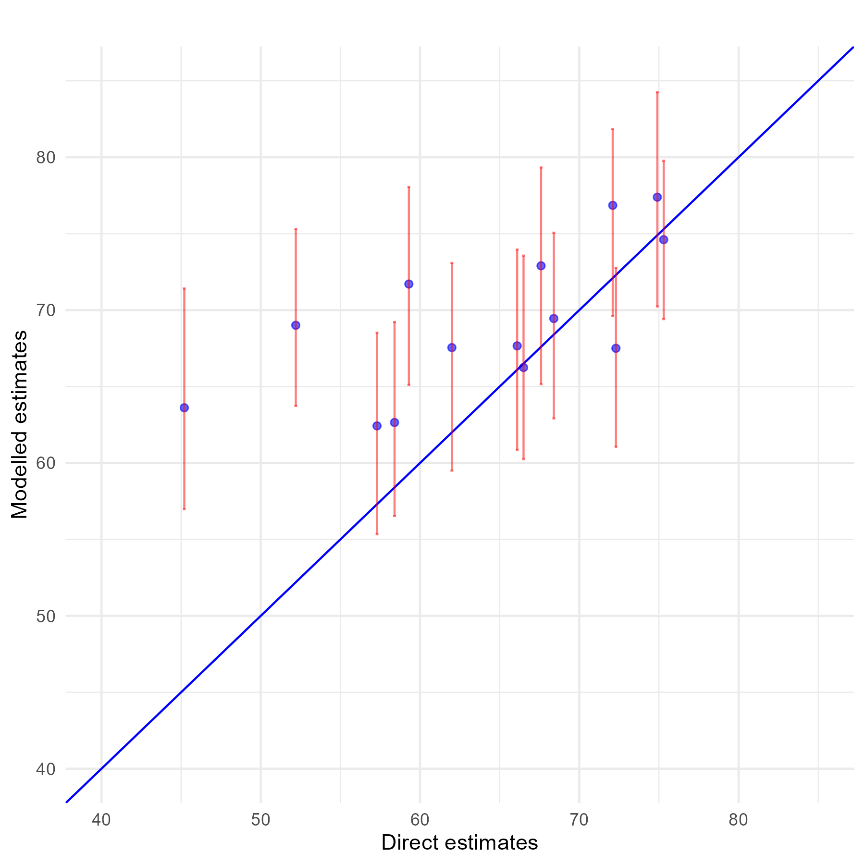


S10 Fig: Direct versus modelled estimates of MCV1 coverage for age group 12-23 months.

Supplement: S10 Fig — (DOCX) [file pcbi.1013989.s010.docx]
