# Supplementary material for: An age-structured spatially varying coefficient model for high-resolution mapping of vaccination coverage
Source: PLoS Comput Biol. 2026 Feb 17;22(2):e1013989. doi: 10.1371/journal.pcbi.1013989 (PMC12928601; doi:10.1371/journal.pcbi.1013989)
Supplement: S5 Fig — (DOCX) [file pcbi.1013989.s005.docx]

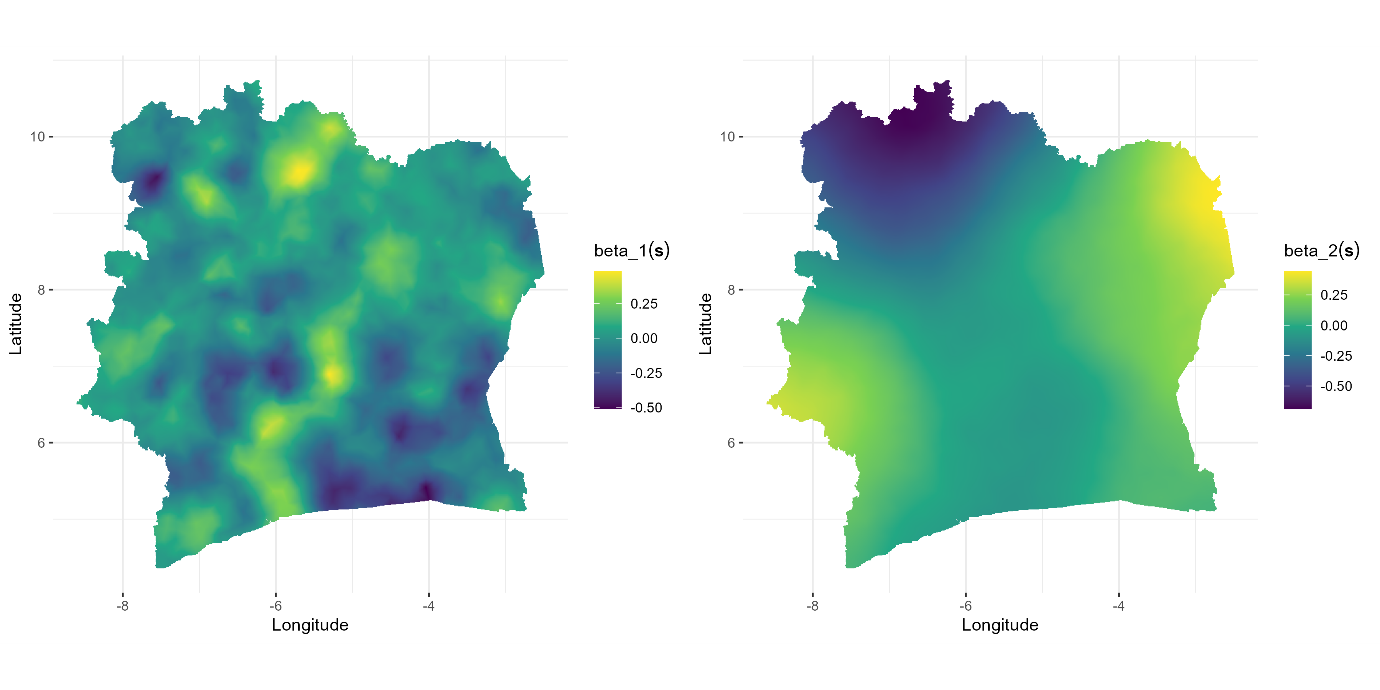


S5 Fig: Maps of $\beta_{1}(\boldsymbol{s})$ and $\beta_{2}(\boldsymbol{s})$ capturing random spatial adjustments to differences in (the log-odds of) vaccination between the 9-11 month age group and each of 12-23 month and 24-35 month age groups, respectively, estimated using MODsvc1.
