# Supplementary material for: An age-structured spatially varying coefficient model for high-resolution mapping of vaccination coverage
Source: PLoS Comput Biol. 2026 Feb 17;22(2):e1013989. doi: 10.1371/journal.pcbi.1013989 (PMC12928601; doi:10.1371/journal.pcbi.1013989)
Supplement: S6 Fig — (DOCX) [file pcbi.1013989.s006.docx]

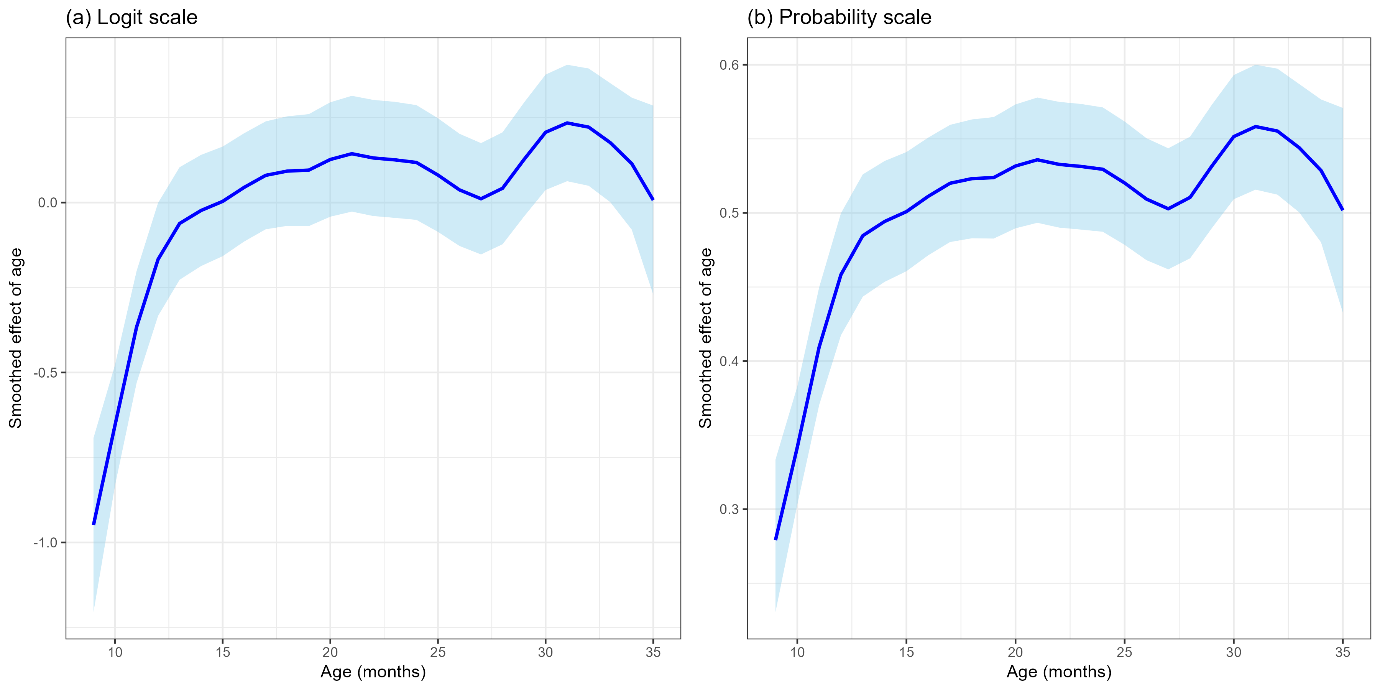


S6 Fig: Plots of the estimated smooth function of age and associated uncertainties using MODsmooth on the (a) logit (i.e., log-odds) and (b) probability scales.
