# Supplementary material for: An age-structured spatially varying coefficient model for high-resolution mapping of vaccination coverage
Source: PLoS Comput Biol. 2026 Feb 17;22(2):e1013989. doi: 10.1371/journal.pcbi.1013989 (PMC12928601; doi:10.1371/journal.pcbi.1013989)
Supplement: S7 Fig — (DOCX) [file pcbi.1013989.s007.docx]

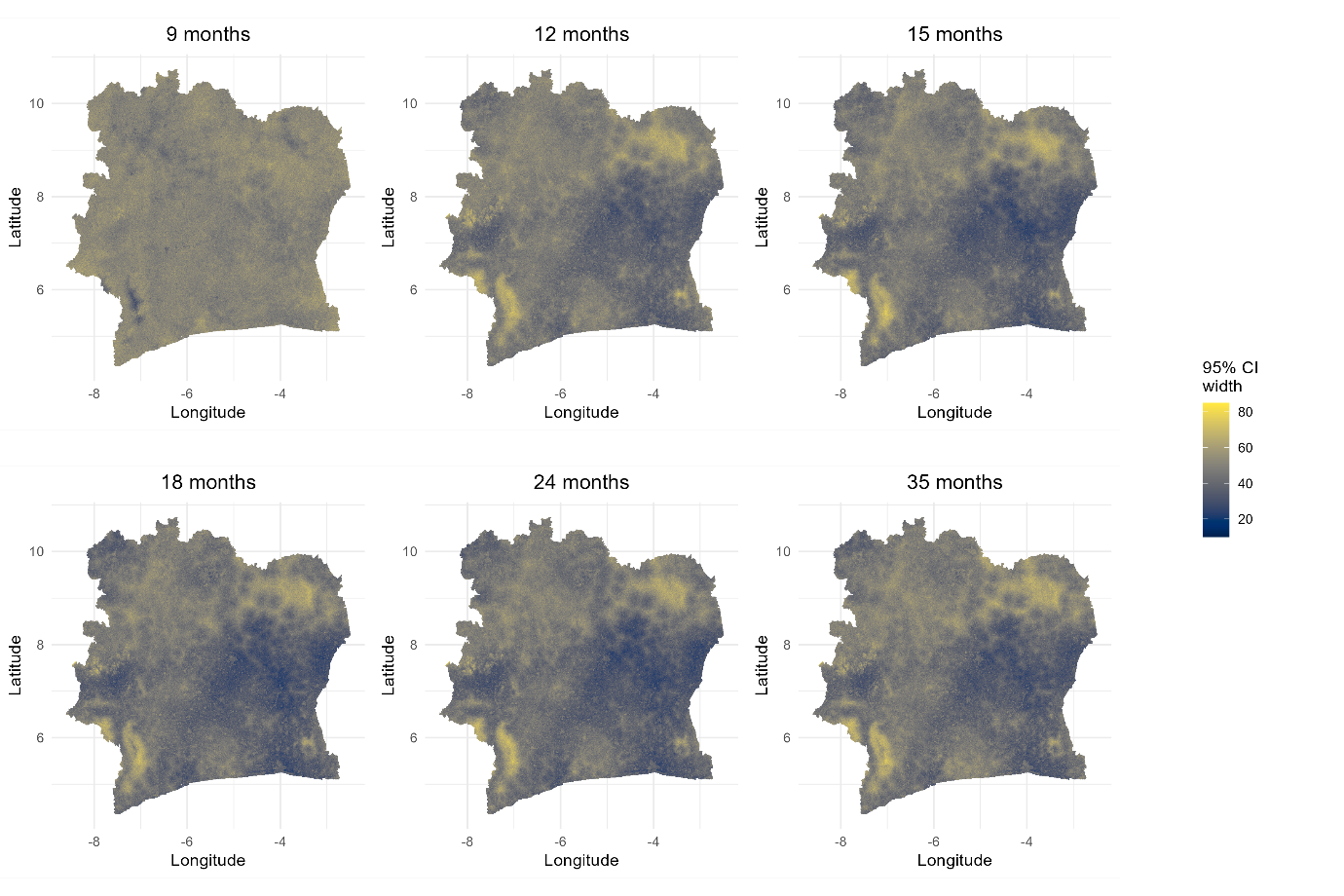


S7 Fig: Uncertainty maps (95% credible interval (CI) widths) associated with 1x1 km estimates of MCV1 coverage for single age points.
