# Supplementary material for: An age-structured spatially varying coefficient model for high-resolution mapping of vaccination coverage
Source: PLoS Comput Biol. 2026 Feb 17;22(2):e1013989. doi: 10.1371/journal.pcbi.1013989 (PMC12928601; doi:10.1371/journal.pcbi.1013989)
Supplement: S8 Fig — The plots have been ordered according to the estimated coverage in the 9-11 month age group. (DOCX) [file pcbi.1013989.s008.docx]

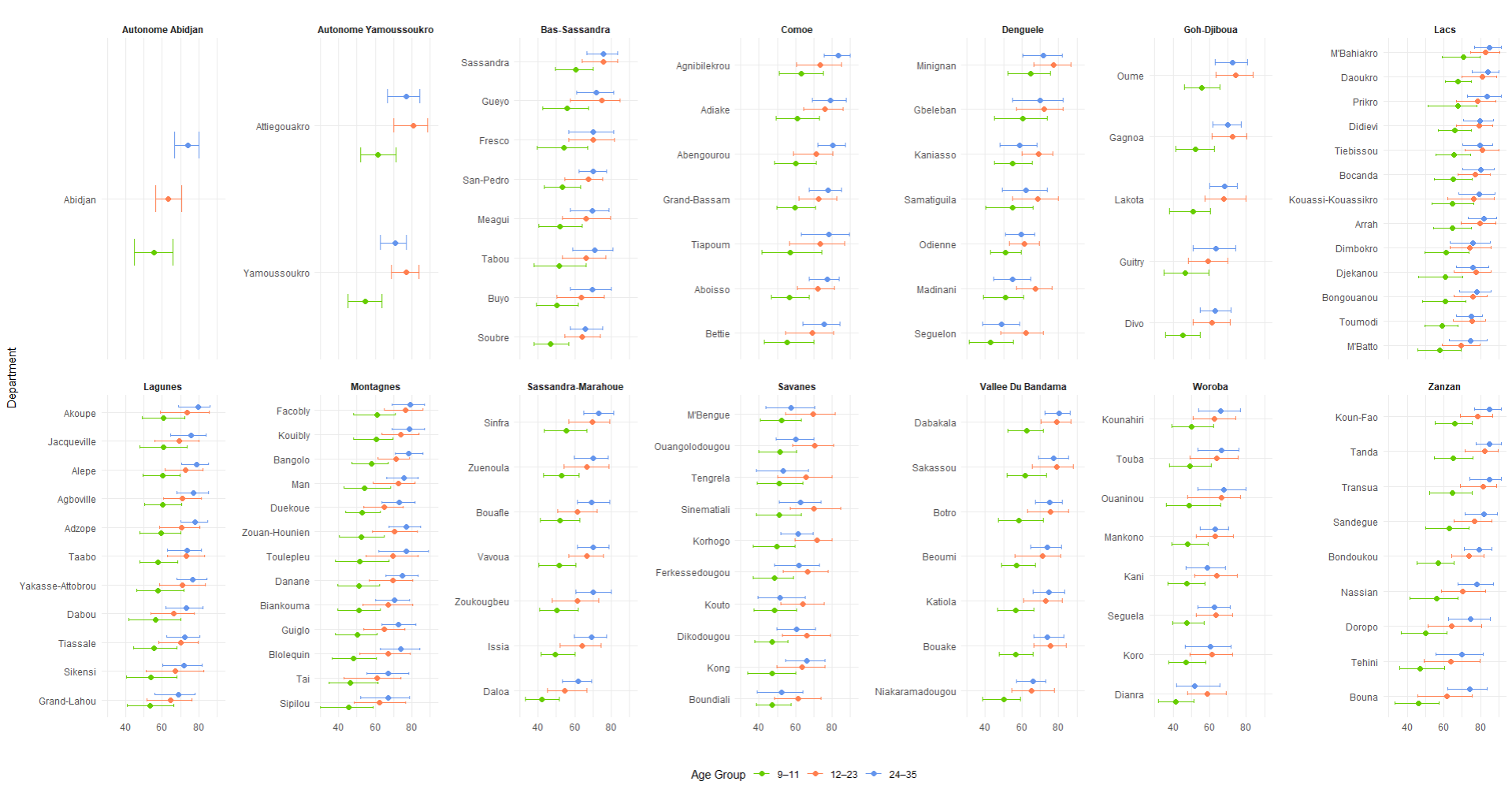


S8 Fig: Department level estimates of MCV1 coverage and associated uncertainties (95% credible intervals) for each of the 14 districts of Cote d’Ivoire. The plots have been ordered according to the estimated coverage in the 9-11 month age group.
