# Supplementary material for: An age-structured spatially varying coefficient model for high-resolution mapping of vaccination coverage
Source: PLoS Comput Biol. 2026 Feb 17;22(2):e1013989. doi: 10.1371/journal.pcbi.1013989 (PMC12928601; doi:10.1371/journal.pcbi.1013989)
Supplement: S9 Fig — Boundary data used in these plots were obtained from geoBoundaries (www.geoboundaries.org) and are available under a CC BY 4.0 license. (DOCX) [file pcbi.1013989.s009.docx]

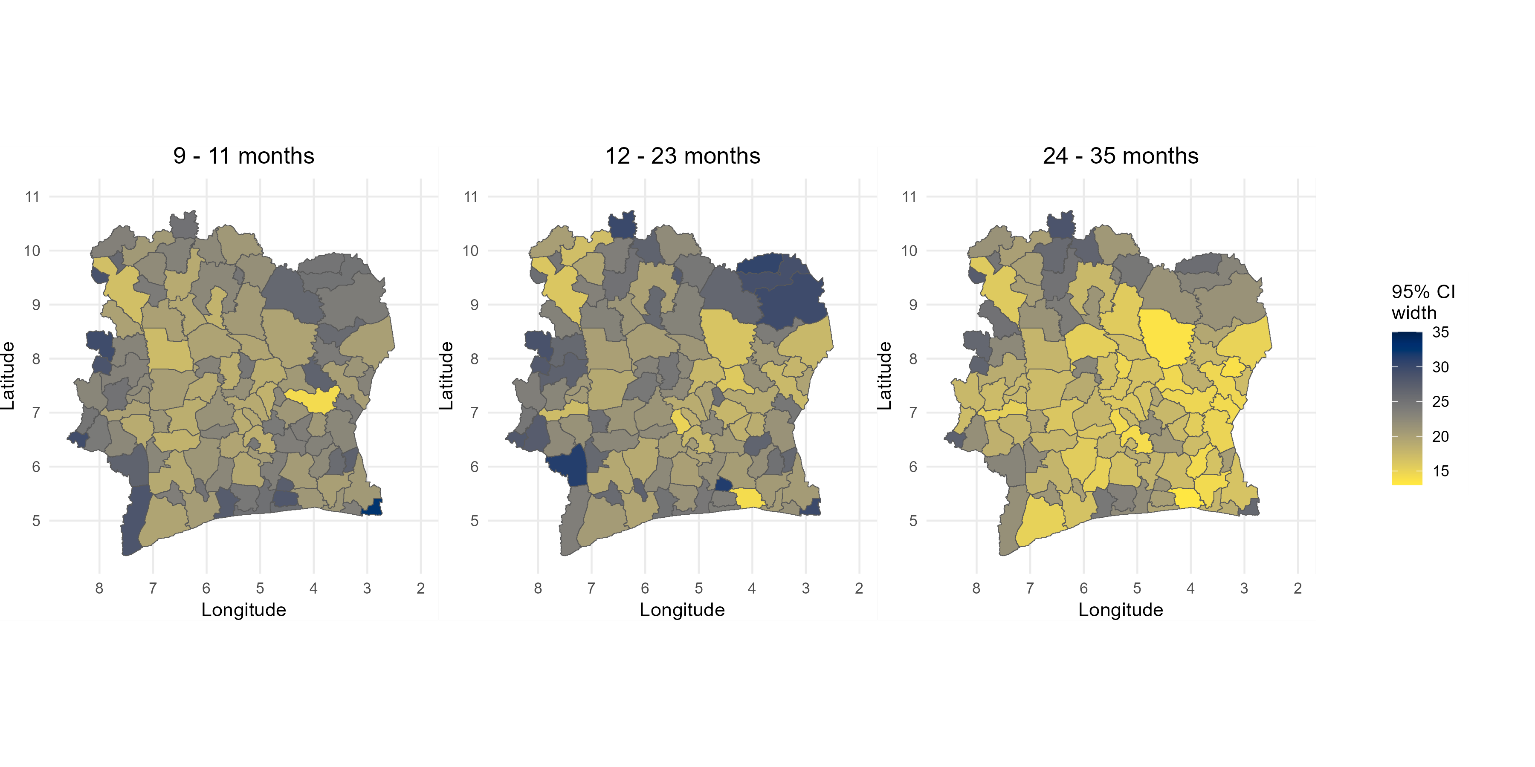


Figure 9: Uncertainty (95% credible interval width) maps for department level estimates of MCV1 coverage for all three age groups. Boundary data used in these plots were obtained from geoBoundaries ([www.geoboundaries.org](http://www.geoboundaries.org)) and are available under a CC BY 4.0 license.
