# Supplementary material for: An age-structured spatially varying coefficient model for high-resolution mapping of vaccination coverage
Source: PLoS Comput Biol. 2026 Feb 17;22(2):e1013989. doi: 10.1371/journal.pcbi.1013989 (PMC12928601; doi:10.1371/journal.pcbi.1013989)
Supplement: S1 Table — Covariates 1 – 9 are the externally sourced geospatial covariates while covariates 10 – 13 are the DHS-derived covariates. (DOCX) [file pcbi.1013989.s012.docx]

S1 Table: Descriptions of the geospatial covariates used in the study. Covariates 1 – 9 are the externally sourced geospatial covariates while covariates 10 – 13 are the DHS-derived covariates.

| S/N | Covariate | Description | Year | Source |
| --- | --- | --- | --- | --- |
| Externally sourced geospatial covariates | | | | |
| 1 | Urban and rural areas | Urban and rural areas produced using WorldPop 2018 unconstrained total population estimates and information obtained from the 2021 Cote d’Ivoire DHS as described in Utazi et al (2022) | 2021 | WorldPop (www.worldpop.org - School of Geography and Environmental Science, University of Southampton; Department of Geography and Geosciences, University of Louisville; Departement de Geographie, Universite de Namur) and Center for International Earth Science Information Network (CIESIN), Columbia University (2018). Global High Resolution Population Denominators Project - Funded by The Bill and Melinda Gates Foundation (OPP1134076). <https://dx.doi.org/10.5258/SOTON/WP00670>  Institut National de la Statistique-INS et ICF, Demographic and Health Surveys of Côte d'Ivoire, 2021- Final Report, INS/Côte d'Ivoire & ICF, Available at  [www.dhsprogram.com/pubs/pdf/FR385/FR385.pdf](http://www.dhsprogram.com/pubs/pdf/FR385/FR385.pdf), 2021  Utazi CE et al (2022). Conditional probability and ratio-based approaches for mapping the coverage of multi-dose vaccines. *Statistics in Medicine*; 41(29): 5662-5678. |
| 2 | Vegetation index | MODIS Mid-Infrared Vegetation index 16-day mean | 2017-2021 | Didan, K. (2021). MODIS/Aqua Vegetation Indices 16-Day L3 Global 1km SIN Grid V061 [Data set]. NASA EOSDIS Land Processes DAAC. <https://doi.org/10.5067/MODIS/MYD13A2.061> |
| 3 | Average wet days | Number of wet days (averaged over 2017 – 2021) | 2017-2021 | Harris, I., Osborn, T. J., Jones, P., & et al. (2020). Version 4 of the CRU TS monthly high-resolution gridded multivariate climate dataset. Scientific Data, 7, 109. <https://doi.org/10.1038/s41597-020-0453-3> |
| 4 | Distance to conflict locations | Distance to UCDP conflict locations averaged over 2016 – 2020 (Metres) | 2016-2020 | [Derived from] Uppsala Conflict Data Program, UCDP Conflict Encyclopedia: [www.ucdp.uu.se](https://ucdp.uu.se/), Uppsala University. |
| 5 | Elevation | Calculation of elevation above the sea level (Metres) | 2020 | https://www.viewfinderpanoramas.org/dem3.html |
| 6 | Access to urban areas | Estimated travel access to urban areas per 1km pixel resolution | 2015 | Weiss, D.J. et al. (2018). A global map of travel time to cities to access inequalities in accessibility in 2015. *Nature 553*(7688):333‐336. doi:10.1038/nature25181. |
| 7 | Travel time to the nearest health facility | Travel time walking (in minutes) from each km^2^ grid to the nearest health facility created using a health facility data base from Maina *et al*. (2019) and the methodology described in Weiss *et al*. (2018) | 2020 | Maina, J. et al. A spatial database of health facilities managed by the public health sector in sub-Saharan Africa. Sci Data 6, 134 (2019). https://doi.org/10.1038/s41597-019-0142-2  Weiss, D.J. et al. (2018). A global map of travel time to cities to access inequalities in accessibility in 2015. *Nature 553*(7688):333‐336. doi:10.1038/nature25181. |
| 8 | Average malaria Prevalence | Malaria parasite prevalence in 2-10-year-olds averaged over 2017– 2020 | 2017-2020 | Weiss, D. J., Lucas, T. C. D., Nguyen, M., Nandi, A. K., Bisanzio, D., et al. (2019). Mapping the global prevalence, incidence, and mortality of Plasmodium falciparum, 2000–17: A spatial and temporal modelling study. The Lancet, 394(10195), 322–331. https://doi.org/10.1016/S0140-6736(19)31097-9 |
| 9 | Average maximum temperature | Maximum temperature (averaged over 2017 – 2021) | 2017-2020 | Harris, I., Osborn, T. J., Jones, P., & et al. (2020). Version 4 of the CRU TS monthly high-resolution gridded multivariate climate dataset. Scientific Data, 7, 109. <https://doi.org/10.1038/s41597-020-0453-3> |
| Cote d’Ivoire DHS-derived geospatial covariates | | | | |
| 10 | Ownership of health card/document | Proportion of children age <= 35 months who owned a vaccination card and/or a health document which were/was seen during the survey | 2021 | Institut National de la Statistique-INS et ICF, Demographic and Health Surveys of Côte d'Ivoire, 2021- Final Report, INS/Côte d'Ivoire & ICF, Available at  [www.dhsprogram.com/pubs/pdf/FR385/FR385.pdf](http://www.dhsprogram.com/pubs/pdf/FR385/FR385.pdf), 2021 |
| 11 | Household wealth | Proportion of households (with at least one living child) belonging to the top three wealth quintiles (middle/richer/richest) | 2021 | Institut National de la Statistique-INS et ICF, Demographic and Health Surveys of Côte d'Ivoire, 2021- Final Report, INS/Côte d'Ivoire & ICF, Available at  [www.dhsprogram.com/pubs/pdf/FR385/FR385.pdf](http://www.dhsprogram.com/pubs/pdf/FR385/FR385.pdf), 2021 |
| 12 | Maternal education | Proportion of mothers who had at least a primary education | 2021 | Institut National de la Statistique-INS et ICF, Demographic and Health Surveys of Côte d'Ivoire, 2021- Final Report, INS/Côte d'Ivoire & ICF, Available at  [www.dhsprogram.com/pubs/pdf/FR385/FR385.pdf](http://www.dhsprogram.com/pubs/pdf/FR385/FR385.pdf), 2021 |
| 13 | Use of media | Proportion of mothers who had access to newspaper/radio/television at least once a week | 2021 | Institut National de la Statistique-INS et ICF, Demographic and Health Surveys of Côte d'Ivoire, 2021- Final Report, INS/Côte d'Ivoire & ICF, Available at  [www.dhsprogram.com/pubs/pdf/FR385/FR385.pdf](http://www.dhsprogram.com/pubs/pdf/FR385/FR385.pdf), 2021 |
