# Supplementary material for: An age-structured spatially varying coefficient model for high-resolution mapping of vaccination coverage
Source: PLoS Comput Biol. 2026 Feb 17;22(2):e1013989. doi: 10.1371/journal.pcbi.1013989 (PMC12928601; doi:10.1371/journal.pcbi.1013989)
Supplement: S2 Table — (DOCX) [file pcbi.1013989.s013.docx]

S2 Table: Brier scores of the fitted models

| Cross-validation type | MODsvc1 | MODsvc2 | MODnosvc | MODsmooth | MODall |
| --- | --- | --- | --- | --- | --- |
| Random | 0.221 | 0.222 | 0.224 | 0.223 | 0.226 |
| Stratified | 0.226 | 0.228 | 0.226 | 0.226 | 0.228 |
