# Supplementary material for: An age-structured spatially varying coefficient model for high-resolution mapping of vaccination coverage
Source: PLoS Comput Biol. 2026 Feb 17;22(2):e1013989. doi: 10.1371/journal.pcbi.1013989 (PMC12928601; doi:10.1371/journal.pcbi.1013989)
Supplement: S3 Table — (DOCX) [file pcbi.1013989.s014.docx]

S3 Table: K-fold cross-validation results for MODall (i.e., for age 9-35 months)

| Cross-validation type | RMSE | MAE | AVG_BIAS | CRPS |
| --- | --- | --- | --- | --- |
| Random | 0.224 | 0.184 | -0.007 | 0.138 |
| Stratified | 0.230 | 0.189 | -0.012 | 0.140 |
