# Supplementary material for: An age-structured spatially varying coefficient model for high-resolution mapping of vaccination coverage
Source: PLoS Comput Biol. 2026 Feb 17;22(2):e1013989. doi: 10.1371/journal.pcbi.1013989 (PMC12928601; doi:10.1371/journal.pcbi.1013989)
Supplement: S4 Table — Estimates corresponding to significant regression coefficients are bolded. (DOCX) [file pcbi.1013989.s015.docx]

S4 Table: Table of parameter estimates for MODsmooth. Estimates corresponding to significant regression coefficients are bolded.

| **Parameter** | **Mean** | **Odds ratio** | **Std. Dev.** | **2.5%** | **97.5%** |
| --- | --- | --- | --- | --- | --- |
| $\hat{\beta}_{0}$ | -2.295 | 0.101 | 5.549 | -13.171 | 8.566 |
| Urban | **-0.584** | 0.558 | **0.143** | **-0.865** | **-0.304** |
| Veg_index | -3.541 | 0.029 | 2.140 | -7.736 | 0.653 |
| Wetdays | -0.097 | 0.908 | 0.103 | -0.298 | 0.104 |
| Dist_conf | 0.150 | 1.162 | 0.089 | -0.024 | 0.324 |
| Elevation | $-6.3\times{10}^{-6}$ | 1.000 | $8.8\times{10}^{-6}$ | $-2.4\times10^-5$ | $1.1\times{10}^{-5}$ |
| Urban_access | $-1.8\times{10}^{-4}$ | 1.000 | $5.1\times{10}^{-4}$ | $-1.2\times10^-3$ | $8.2\times{10}^{-4}$ |
| Walking_tt | **-0.003** | 0.997 | **0.001** | **-0.005** | **-0.001** |
| Mal_prev | -0.206 | 0.814 | 0.830 | -1.833 | 1.420 |
| Max_temp | 0.088 | 1.092 | 0.144 | -0.194 | 0.370 |
| Mat_educ | **1.053** | 2.866 | **0.236** | **0.591** | **1.515** |
| Health_card | **1.814** | 6.135 | **0.302** | **1.222** | **2.406** |
| Media | 0.507 | 1.660 | 0.271 | -0.024 | 1.039 |
| Wealth | -0.114 | 0.892 | 0.247 | -0.598 | 0.370 |
| $\hat{r}$ | 0.859 | - | 0.376 | 0.359 | 1.808 |
| $\hat{\sigma}_{\omega}$ | 0.367 | - | 0.094 | 0.216 | 0.583 |
| $\hat{\sigma}_{z}^{-1}$ | 150.615 | - | 110.025 | 29.155 | 438.147 |
| $\hat{\sigma}_{\epsilon}^{-1}$ | 3.907 | - | 1.142 | 2.127 | 6.582 |
